# Supplementary material for: The IRE1β-mediated unfolded protein response is repressed by the chaperone AGR2 in mucin producing cells
Source: EMBO J. 2023 Dec 18;43(5):719–53. doi: 10.1038/s44318-023-00014-z (PMC10907699; doi:10.1038/s44318-023-00014-z)
Supplement: Supplementary file 1 — Appendix Figures [file 44318_2023_14_MOESM1_ESM.pdf]

Appendix figures

Table of Contents

Appendix Figure S1 - Modification of the endogenous ERN1 locus of Chinese Hamster Ovarian (CHO) cells to introduce IRE1 luminal domain (LD) variants. 2

Appendix Figure S2 - XBP1 splicing and RIDD activity of IRE1 derivatives...........................3

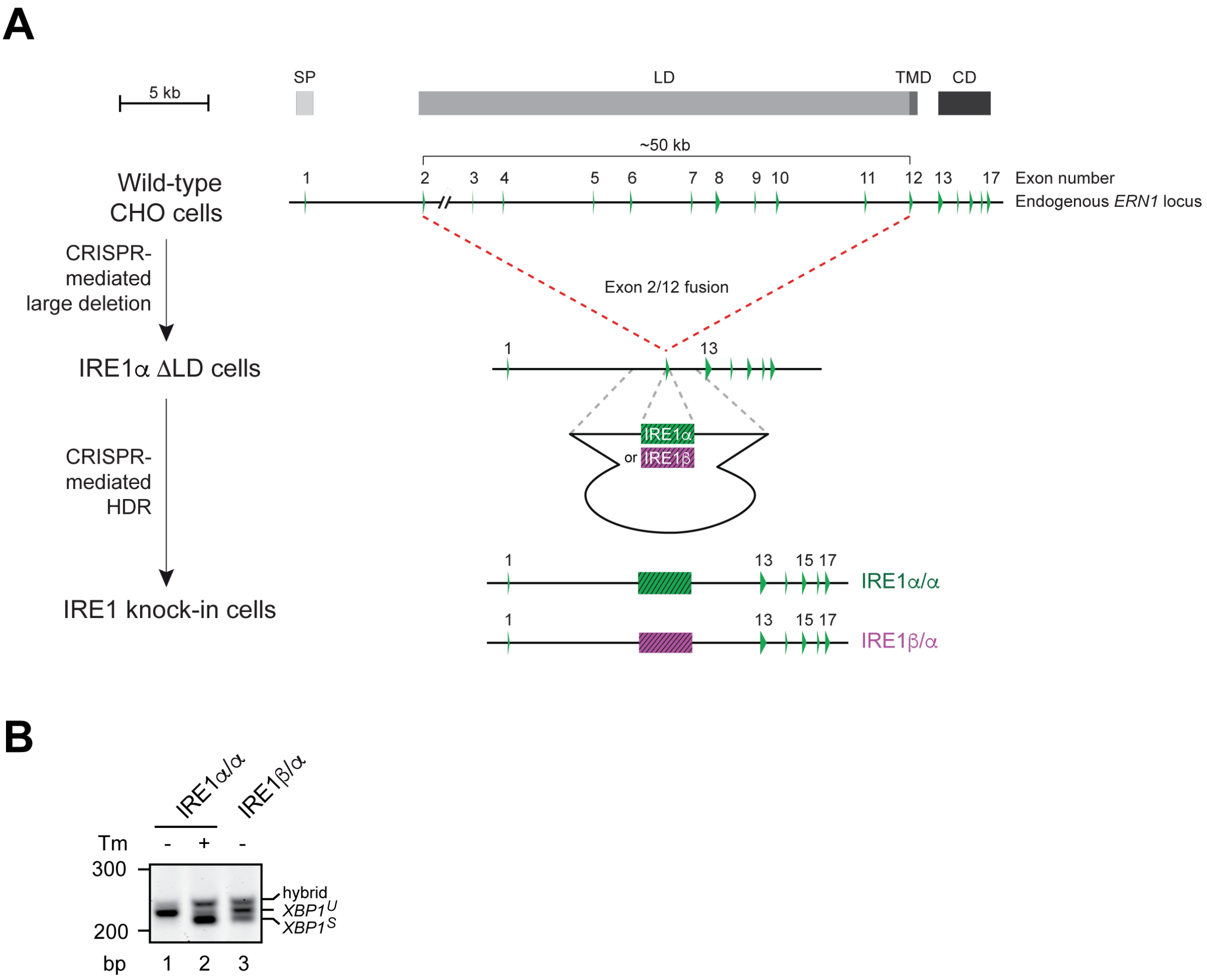

Appendix Figure S1 - Modification of the endogenous *ERN1* locus of Chinese Hamster Ovarian (CHO) cells to introduce IRE1 luminal domain (LD) variants.

A Schematic description of the homologous recombination platform for creating IRE1 encoding alleles at the endogenous *ERN1* locus. On top, the IRE1 domain structure is mapped onto the respective section of the endogenous *ERN*1 locus encoding signal peptide (SP), LD, transmembrane domain (TMD) and cytosolic domain (CD). CRISPR-Cas9 guides generated a large in-frame deletion between exons 2 and 11 in the endogenous *ERN1* locus. In the resultant *ERN1* null cell line [IRE1α ΔLD, previously described in (Kono *et al*, 2017)] the deleted locus was re-targeted by CRISPR-Cas9 as well as a repair templates containing homology arms and a minigene encoding the LD of mouse IRE1α or IRE1β to reconstruct the locus by encoding the indicated chimeric proteins (see **Fig 1A** for schematic depiction).

B Agarose gel of XBP1 cDNA from tunicamycin (Tm)-treated IRE1α/α or IRE1β/α CHO cells. Migration of the unspliced ($XBP1^u$), spliced ($XBP1^s$) and hybrid-stained DNA fragments is indicated.

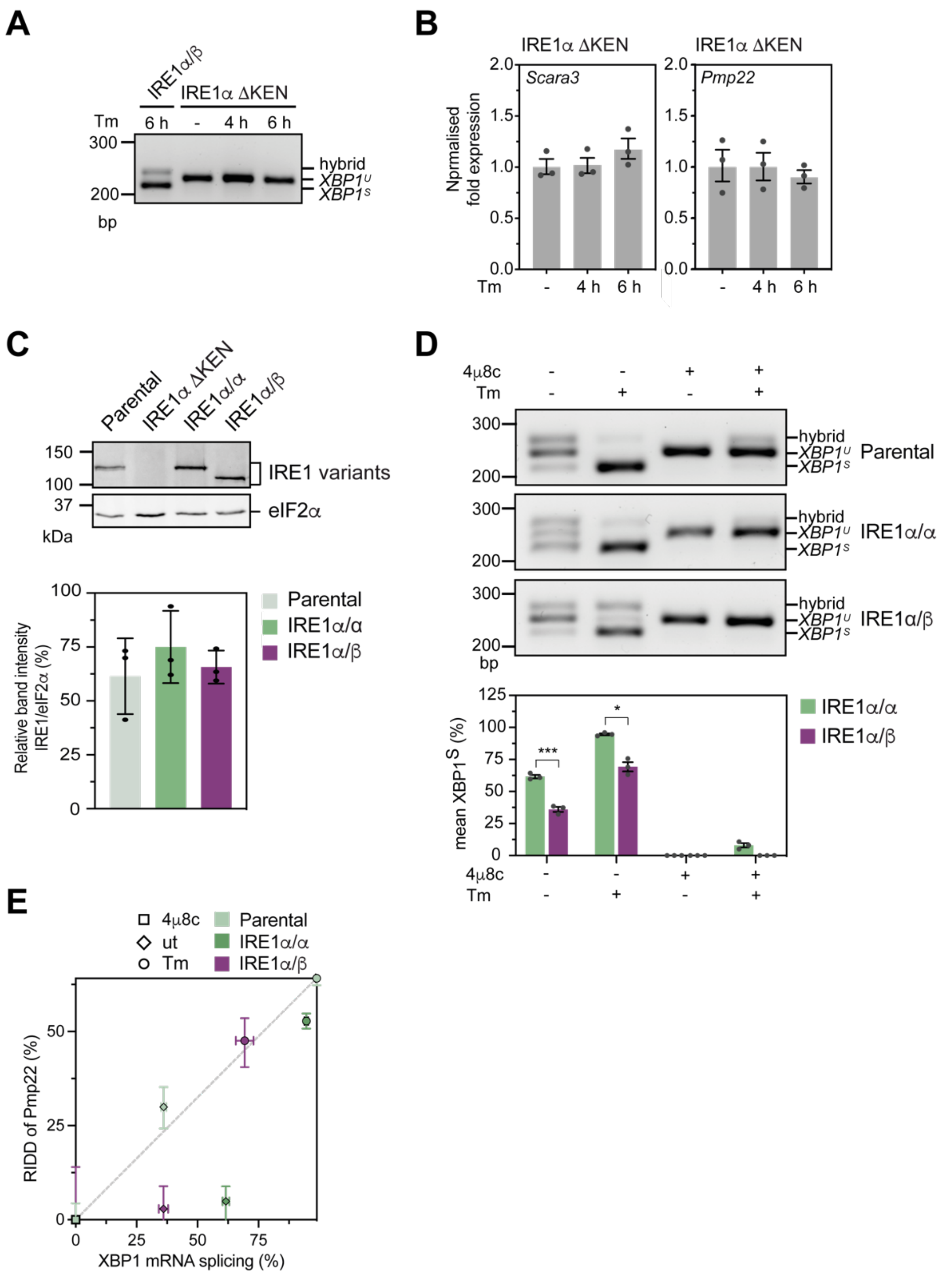

Appendix Figure S2 - XBP1 splicing and RIDD activity of IRE1 derivatives.

A Agarose gel of XBP1 cDNA from tunicamycin (Tm)-treated CHO cells deleted of the IRE1α kinase endonuclease extension domain (IRE1α ΔKEN). Migration of the unspliced (XBP1^{u}),

spliced (XBP1[s]) and hybrid-stained DNA fragments is indicated. A sample from the IRE1α/β ('rescued') cells is included for reference.

B Plot of mRNA abundance of RIDD targets Scara3 and Pmp22 in IRE1α ΔKEN cells [treated as in (A)] as determined by qPCR. The mRNA expression is normalised to untreated cells and was determined as described by (Taylor *et al*, 2019). The bars and error bars represent the geometric mean ± SEM of data obtained from three independent experiments.

C Upper panel: Immunoblot of lysates from cells with the indicated genotype probed with antibodies against IRE1α LD and eIF2α. Lower Panel: Plot of IRE1 protein levels determined by immunoblot analysis and normalised to eIF2α The bars and error bars represent the geometric mean ± SEM of data obtained from three independent experiments.

D Upper panel: Stained DNA fragments of XBP1 cDNA from cells of the indicated genotypes treated with tunicamycin, the IRE1 RNase inhibitor 4μ8c (Cross *et al*, 2012) or both. Lower panel: Plot displaying the fraction of spliced XBP1. The bars and error bars represent the mean ± SEM of data obtained from three independent experiments. Statistical analysis was performed by two-sided unpaired Welch's t test and significance is indicated by asterisks (* $p < 0.05$, *** $p < 0.001$).

E Two-dimensional plot of IRE1 RIDD activity for Pmp22-directed RIDD activity versus the XBP1 mRNA splicing activity of IRE1-inhibited (4μ8c), untreated (ut) and stressed (8 h Tm) parental, CHO IRE1α/α and IRE1α/β cells. Plotted is qPCR data from **Fig 8C** and RT-PCR data from **Appendix Fig S2D**.
