# Supplementary figures and images for: The IRE1β-mediated unfolded protein response is repressed by the chaperone AGR2 in mucin producing cells
Source: EMBO J. 2023 Dec 18;43(5):719–53. doi: 10.1038/s44318-023-00014-z (PMC10907699; doi:10.1038/s44318-023-00014-z)

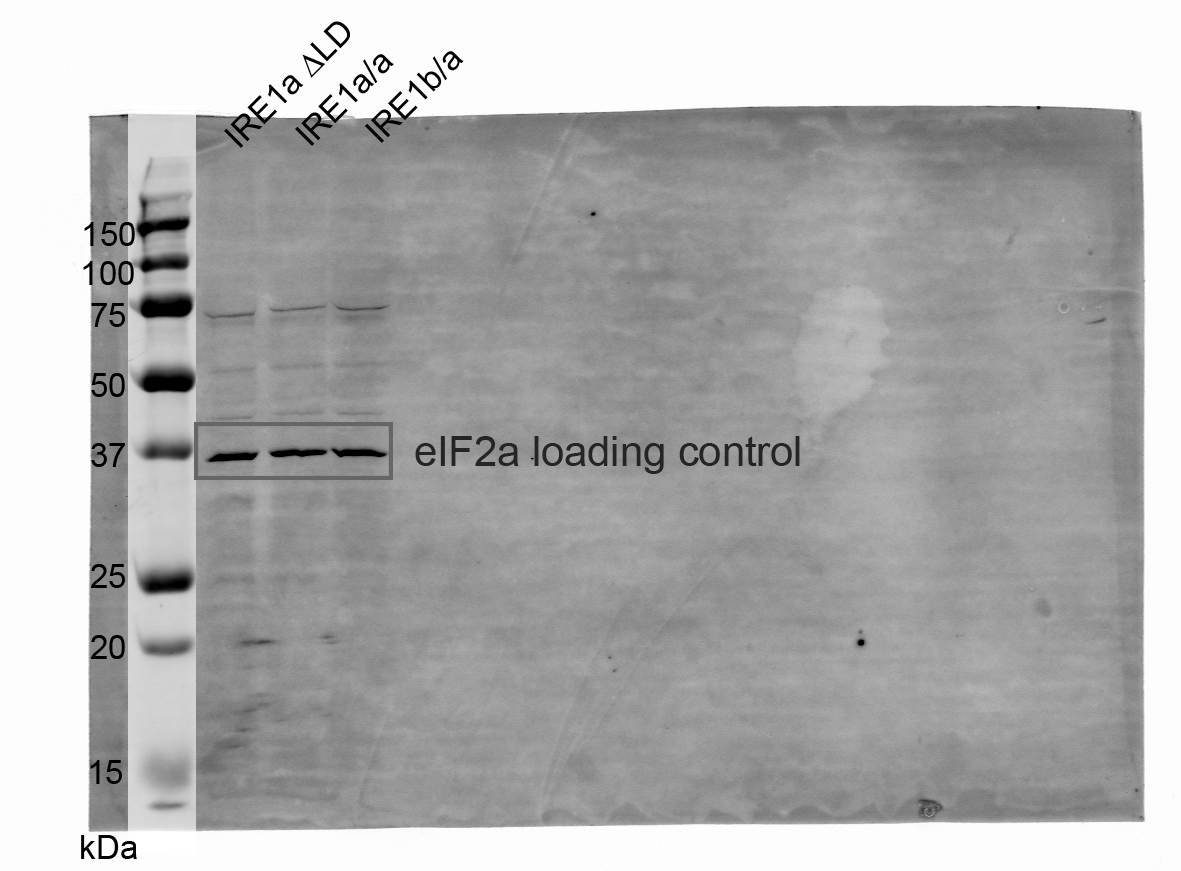

Supplement: Supplementary file 3 — Source Data Fig. 1 [file 44318_2023_14_MOESM3_ESM.zip › Fig 1/D/20220715 MS IP Input/eIF2a loading control.tif]

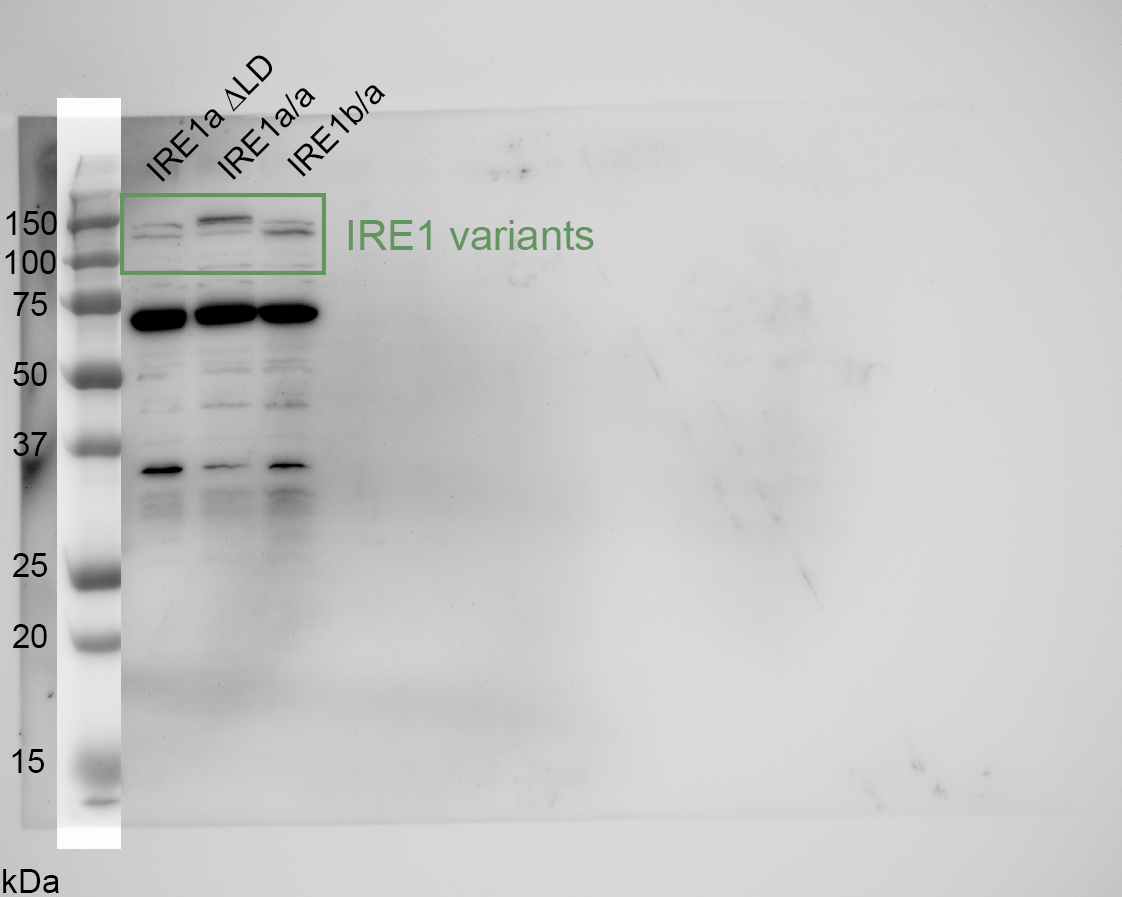

Supplement: Supplementary file 3 — Source Data Fig. 1 [file 44318_2023_14_MOESM3_ESM.zip › Fig 1/D/20220715 MS IP Input/IRE1 variants (detected via IRE1a cytosolic domain).tif]

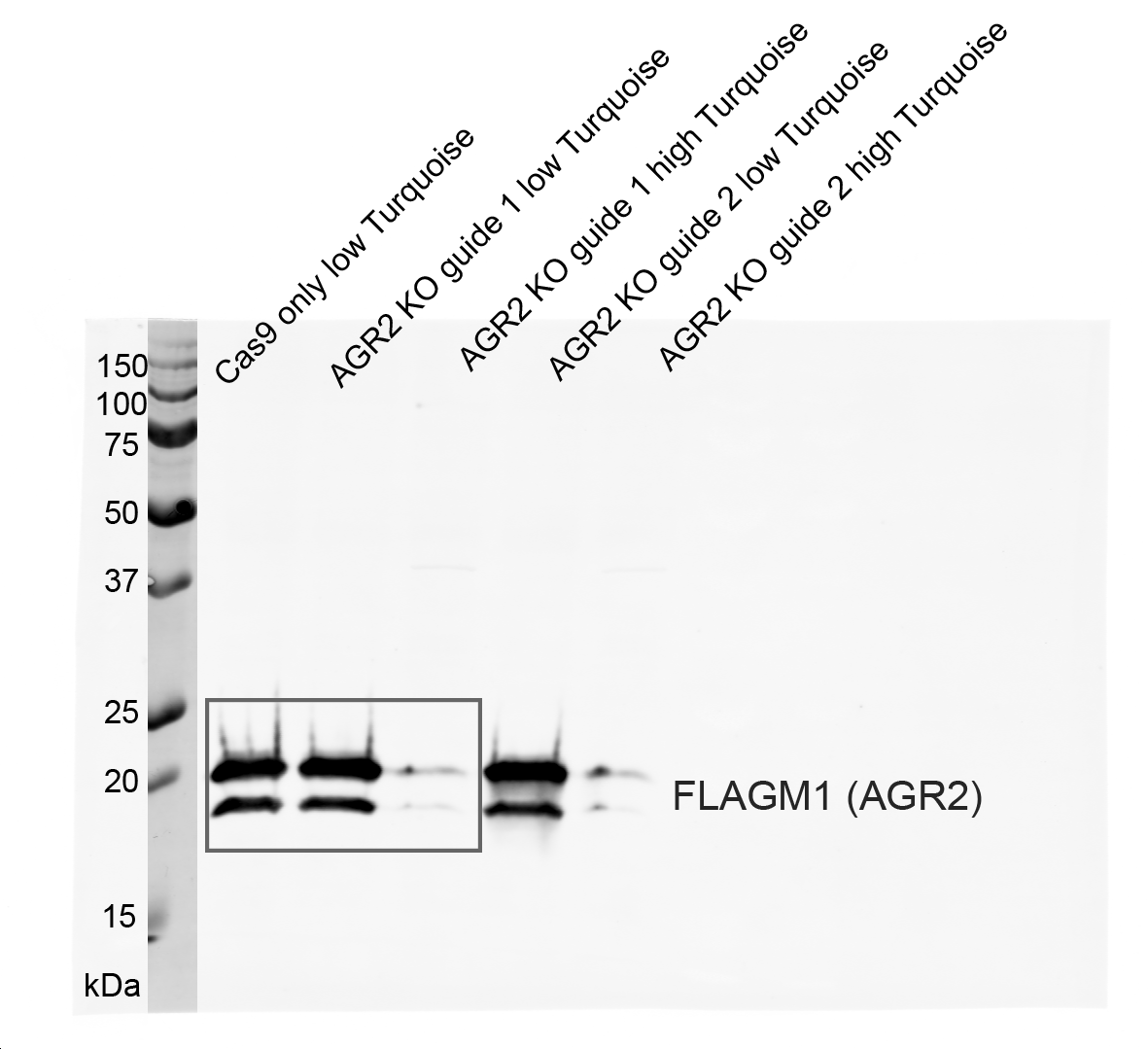

Supplement: Supplementary file 4 — Source Data Fig. 2 [file 44318_2023_14_MOESM4_ESM.zip › Fig 2/E/Western Blot/20210922 FLAGM1 (AGR2).tif]

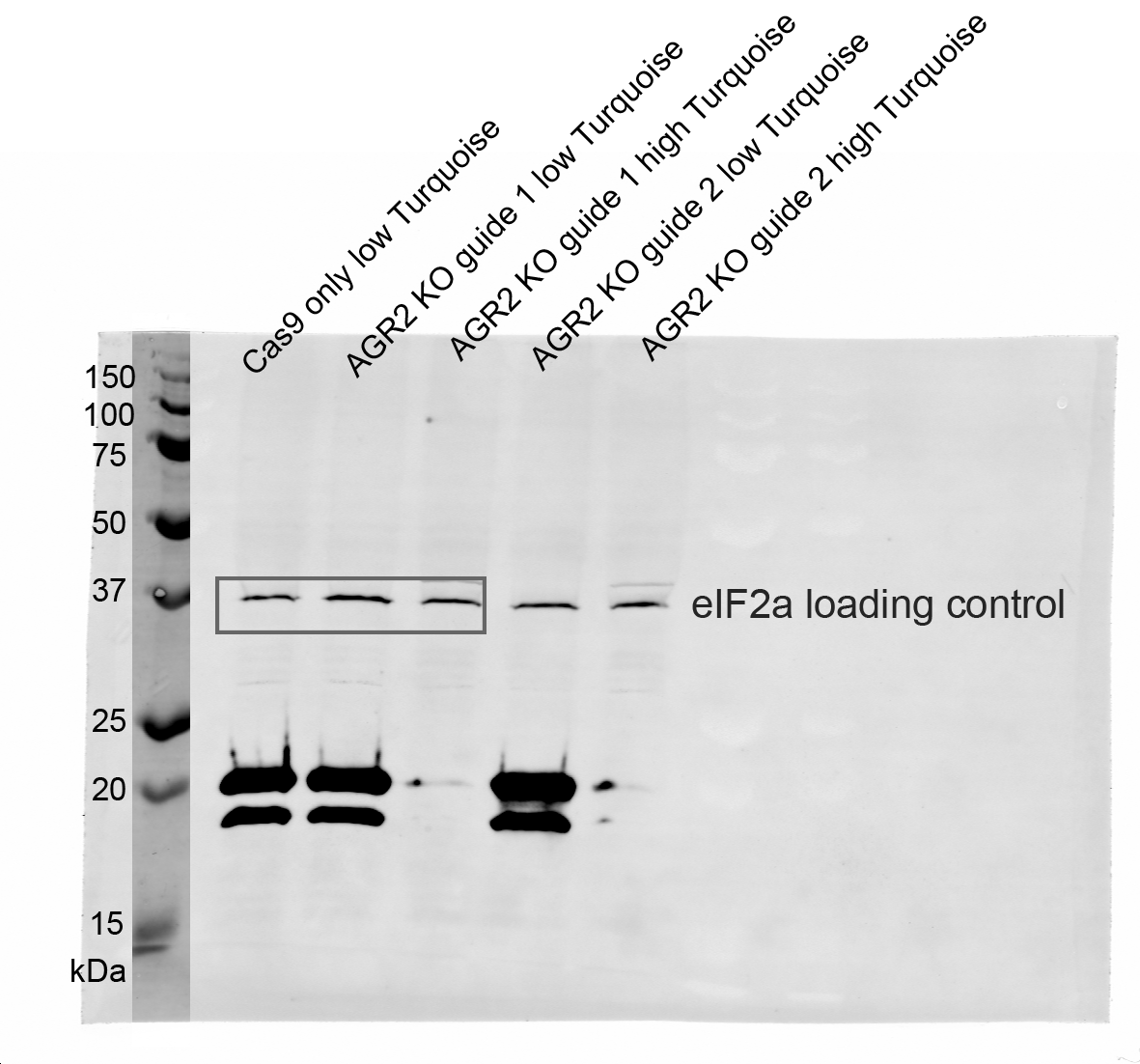

Supplement: Supplementary file 4 — Source Data Fig. 2 [file 44318_2023_14_MOESM4_ESM.zip › Fig 2/E/Western Blot/20210923 eIF2a loading control.tif]

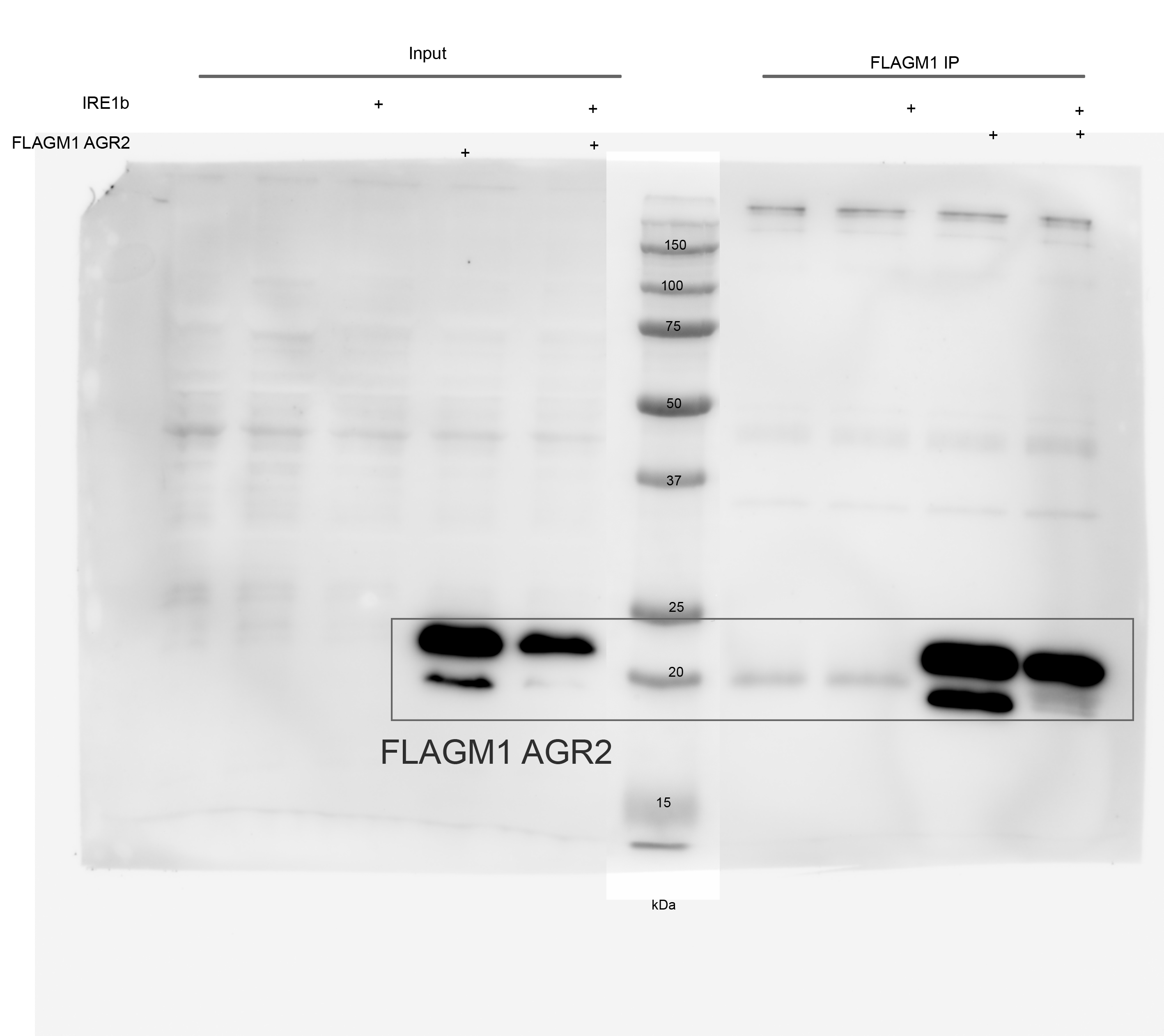

Supplement: Supplementary file 5 — Source Data Fig. 3 [file 44318_2023_14_MOESM5_ESM.zip › Fig 3/C/20220902 rep1/FLAGM1 non-reduced SDS-PAGE.tif]

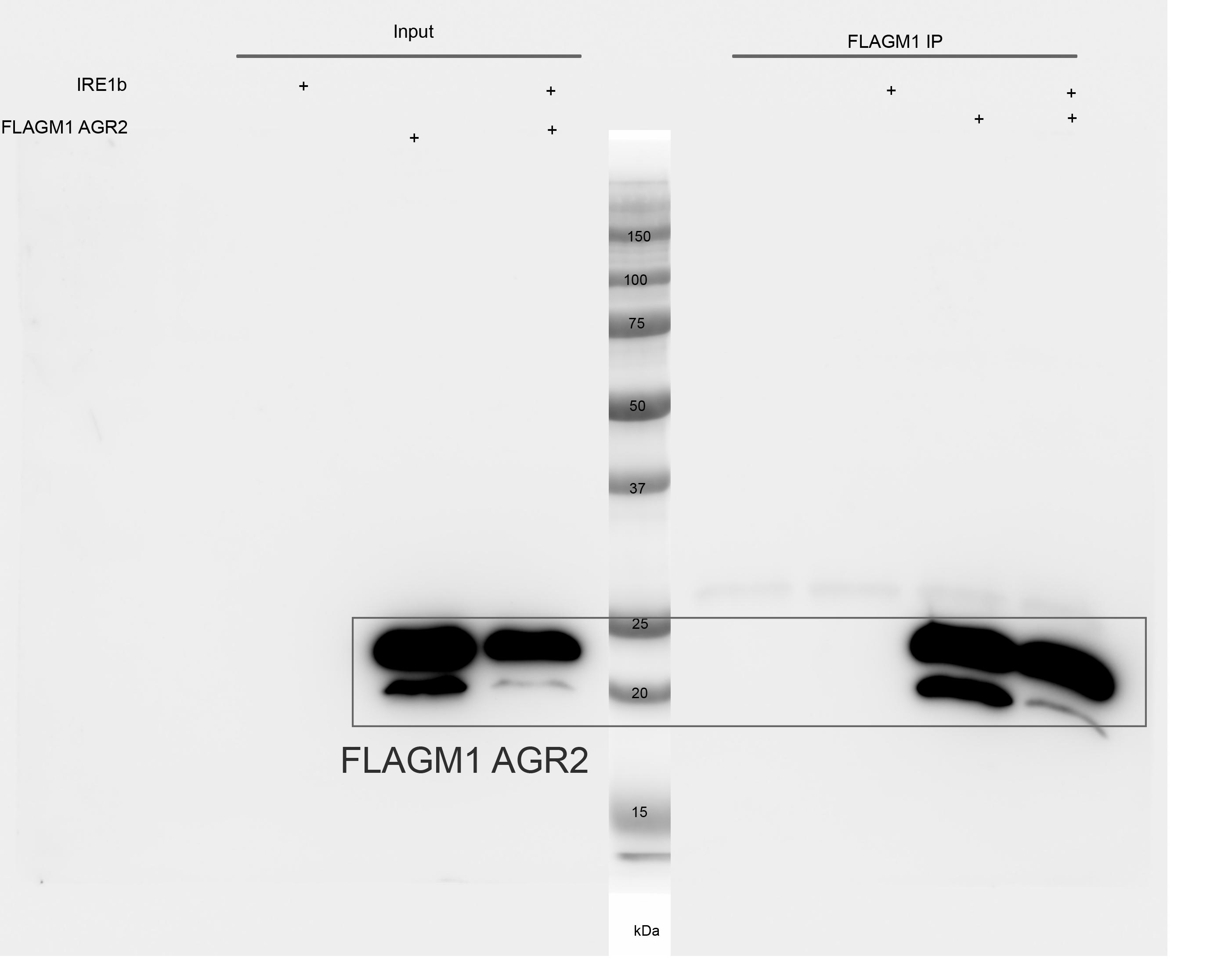

Supplement: Supplementary file 5 — Source Data Fig. 3 [file 44318_2023_14_MOESM5_ESM.zip › Fig 3/C/20220902 rep1/FLAGM1 reduced SDS-PAGE.tif]

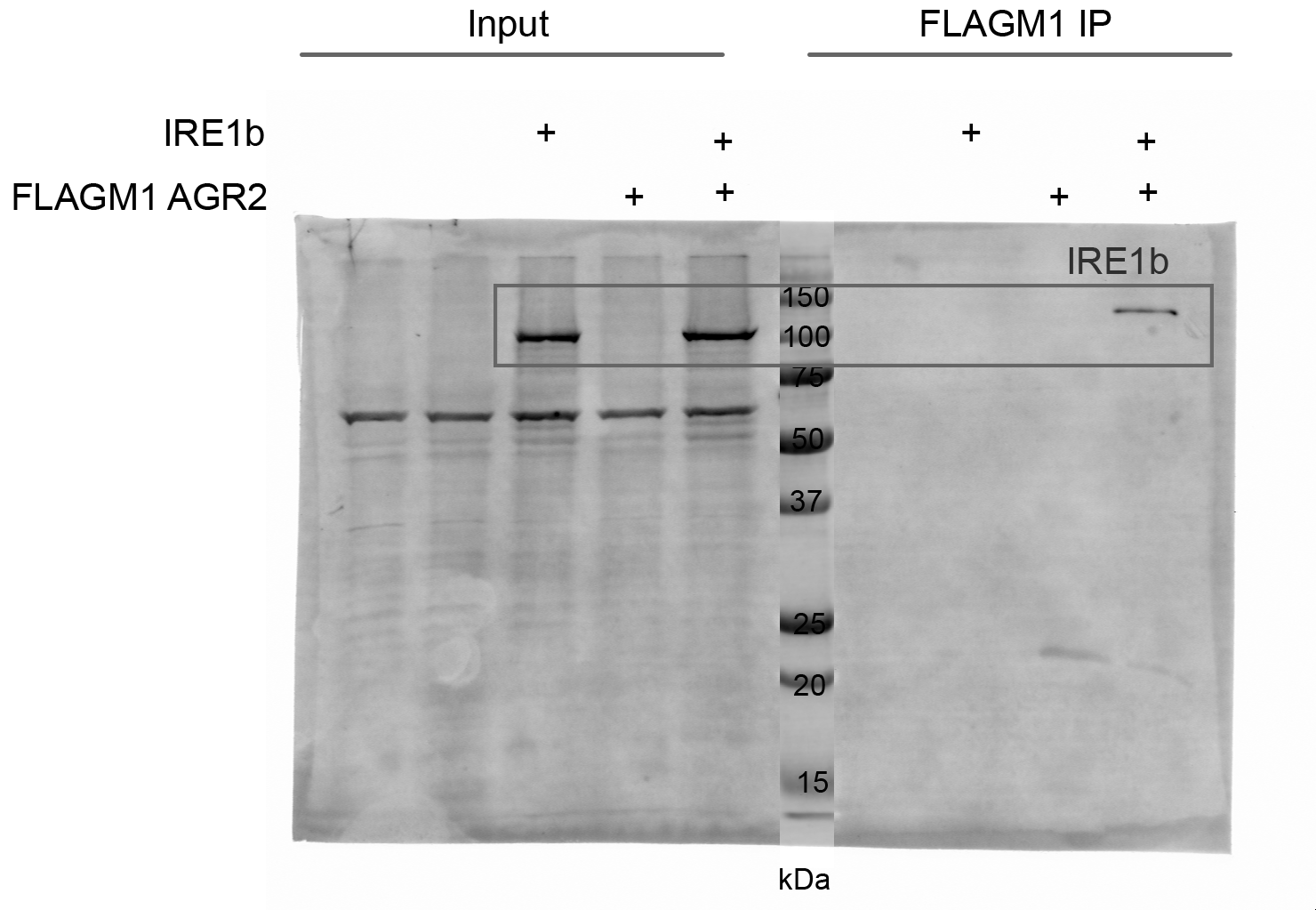

Supplement: Supplementary file 5 — Source Data Fig. 3 [file 44318_2023_14_MOESM5_ESM.zip › Fig 3/C/20220902 rep1/N109 non-reduced SDS-PAGE.tif]

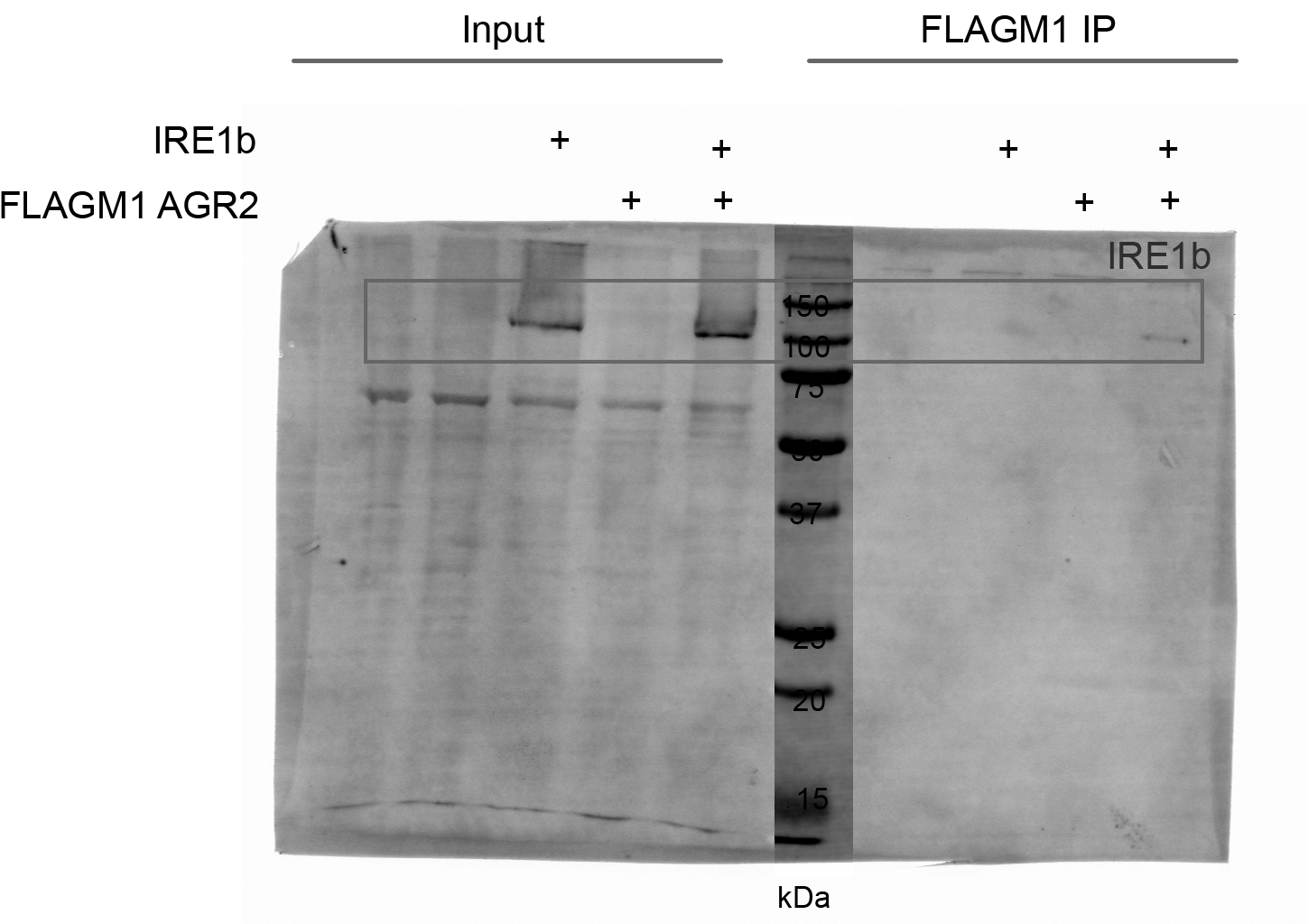

Supplement: Supplementary file 5 — Source Data Fig. 3 [file 44318_2023_14_MOESM5_ESM.zip › Fig 3/C/20220902 rep1/N109 reduced SDS-PAGE.tif]

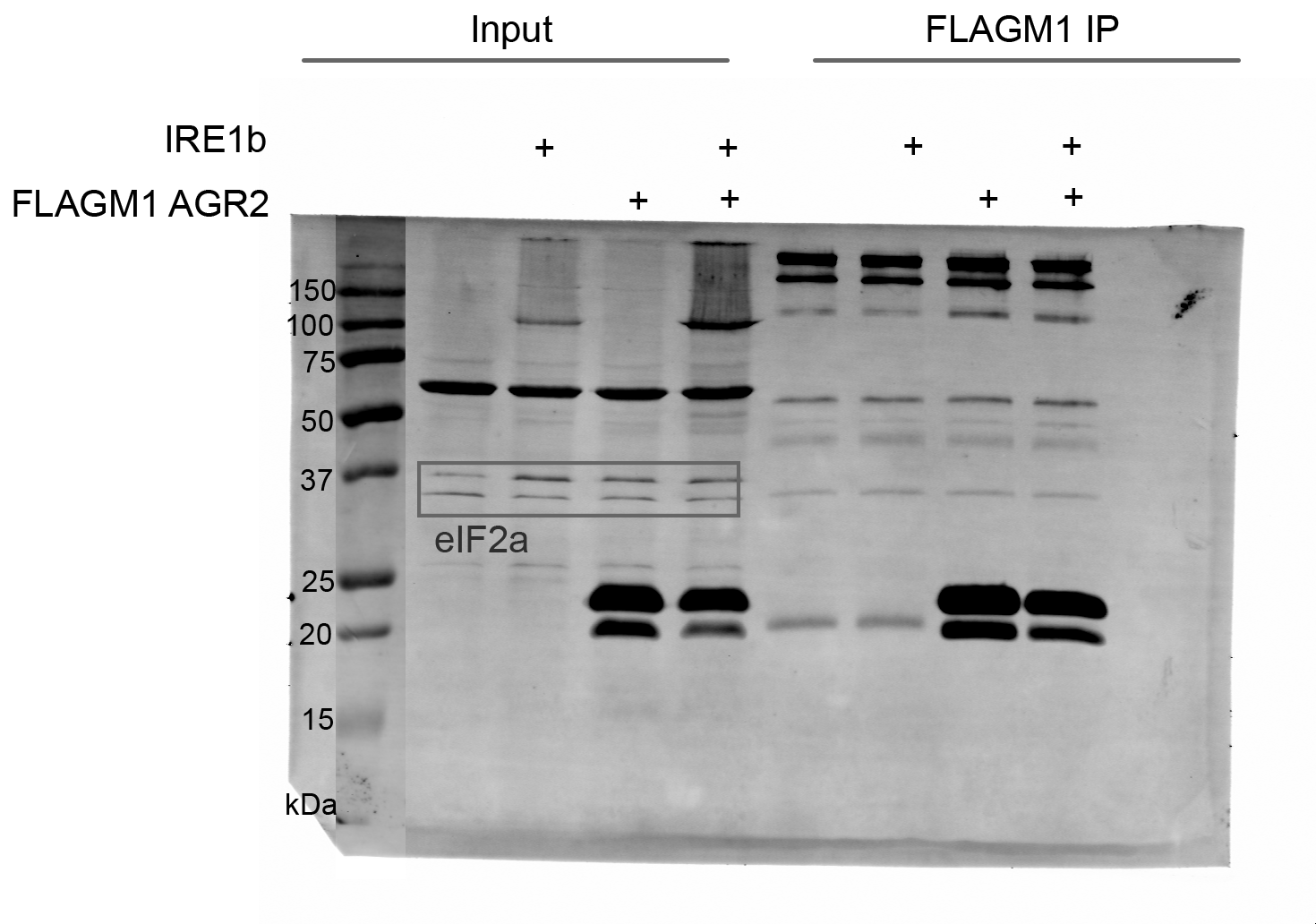

Supplement: Supplementary file 5 — Source Data Fig. 3 [file 44318_2023_14_MOESM5_ESM.zip › Fig 3/C/20220926 rep2 (depicted in the Fig)/eIF2a non-reduced SDS-PAGE.tif]

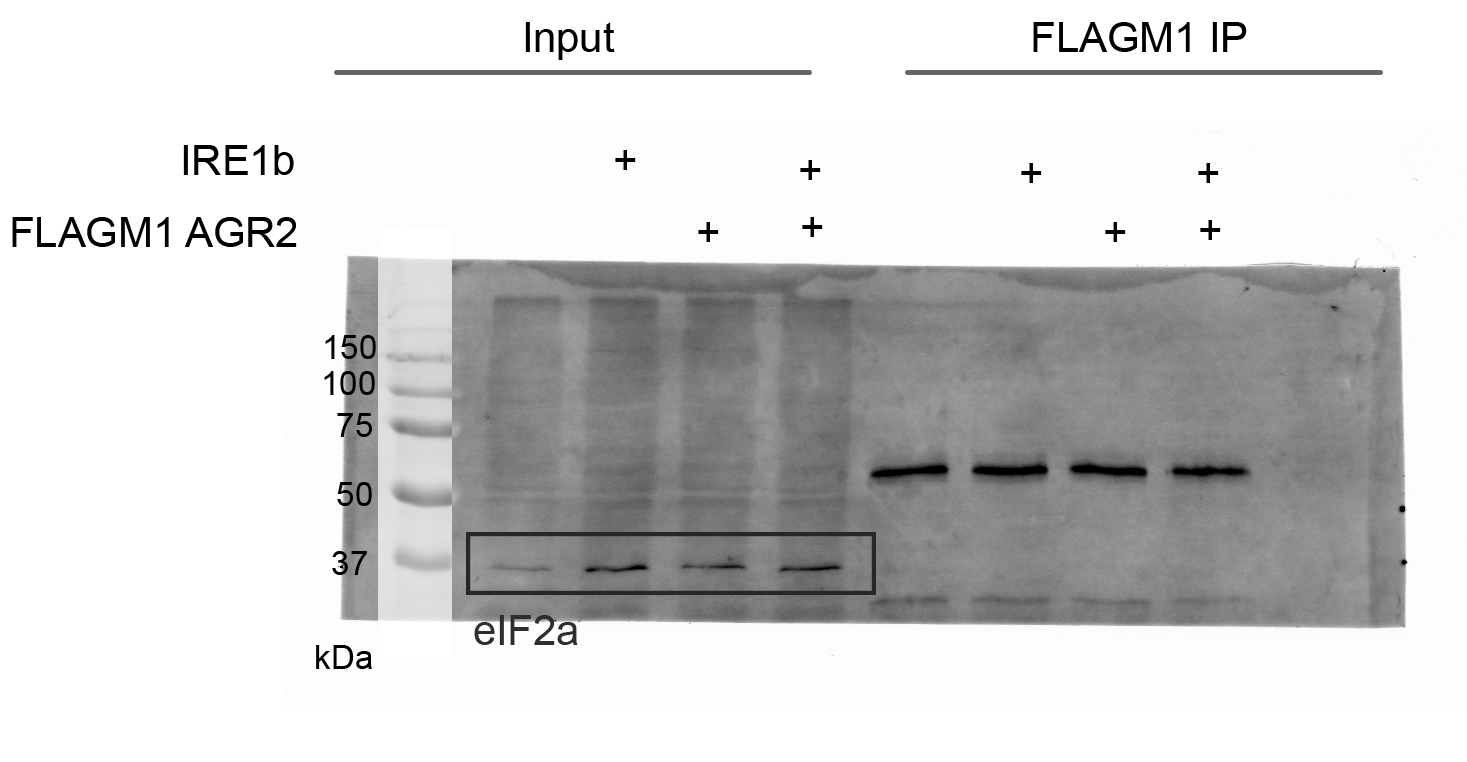

Supplement: Supplementary file 5 — Source Data Fig. 3 [file 44318_2023_14_MOESM5_ESM.zip › Fig 3/C/20220926 rep2 (depicted in the Fig)/eIF2a reduced SDS-PAGE.tif]

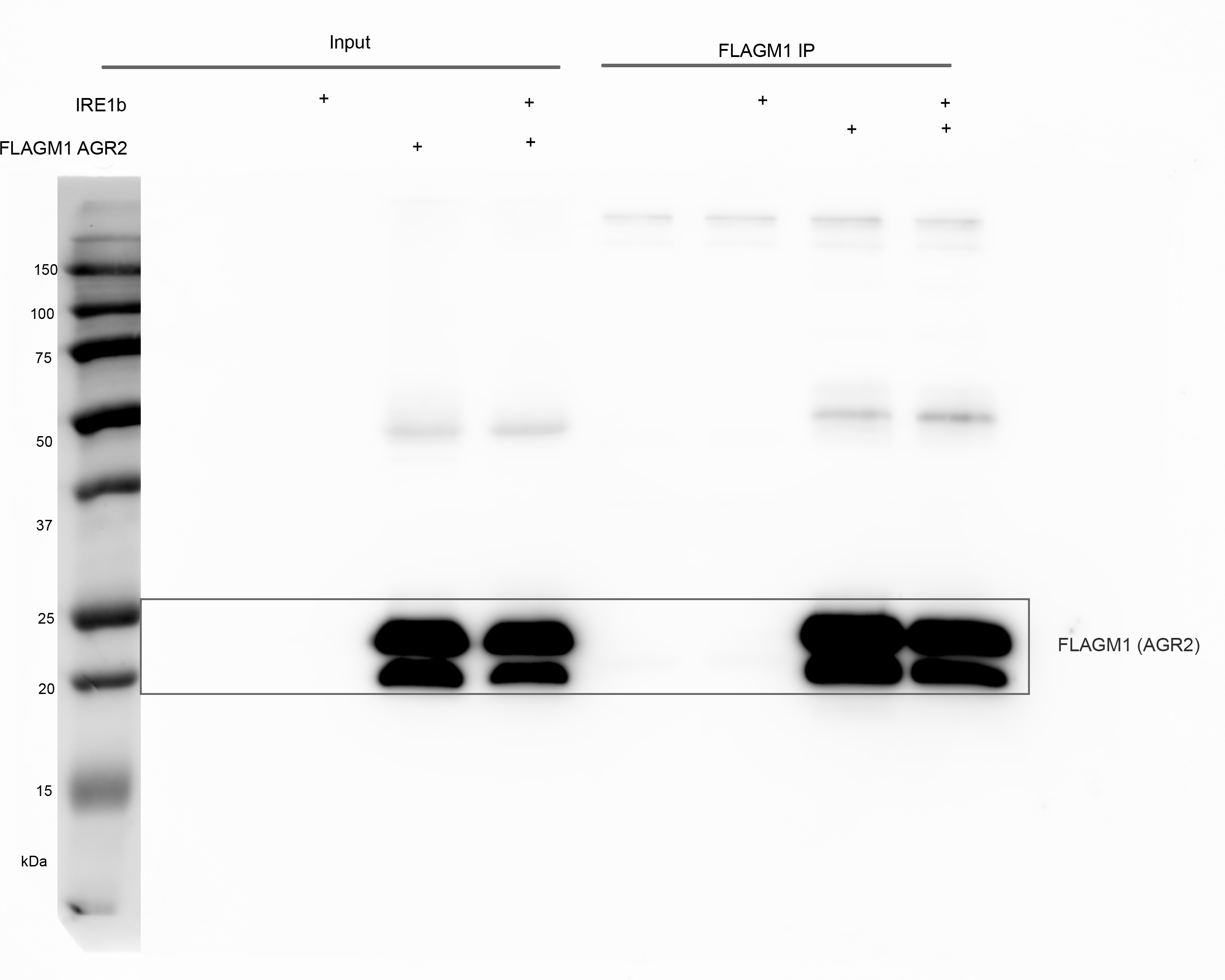

Supplement: Supplementary file 5 — Source Data Fig. 3 [file 44318_2023_14_MOESM5_ESM.zip › Fig 3/C/20220926 rep2 (depicted in the Fig)/FLAGM1 non-reduced SDS-PAGE.tif]

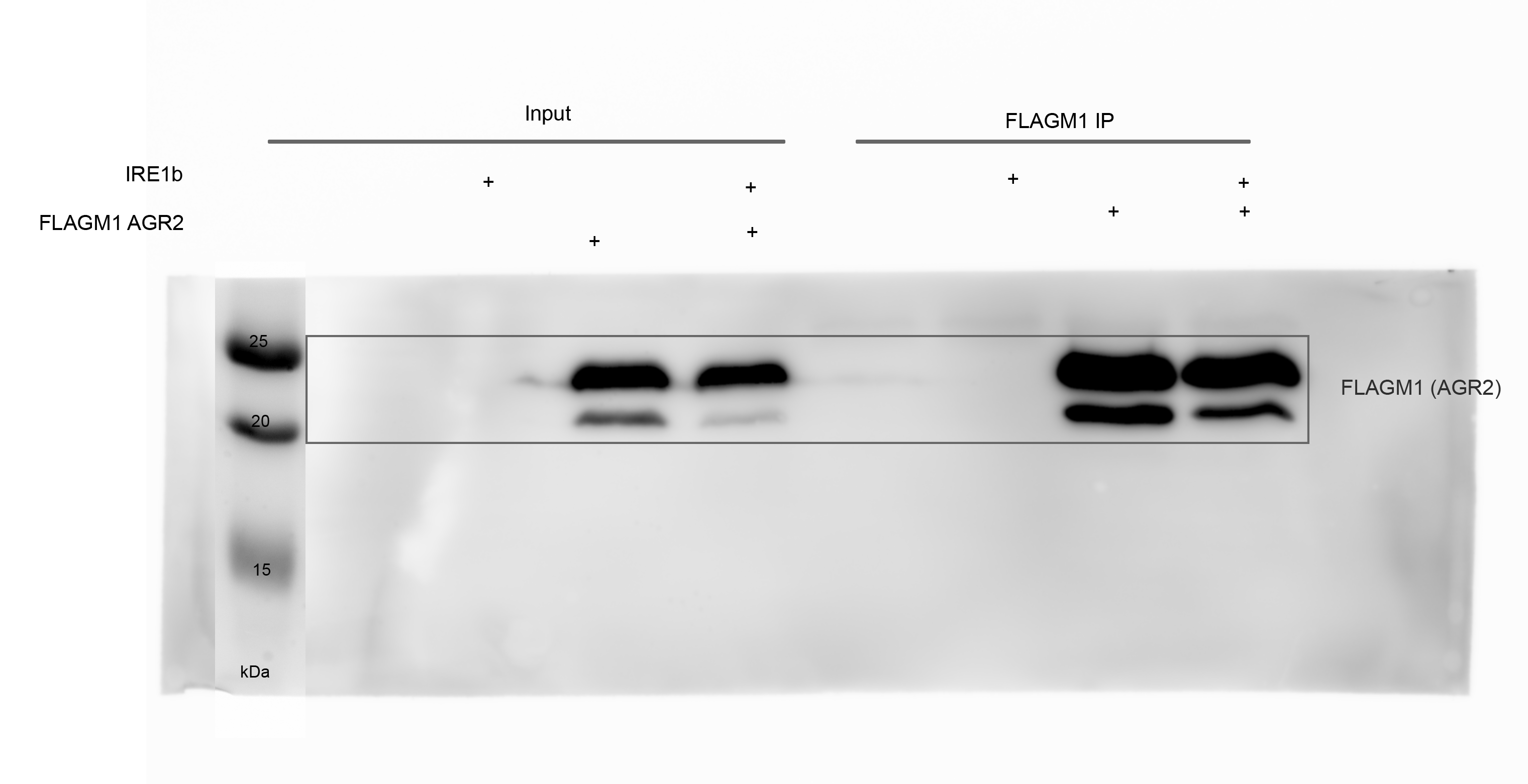

Supplement: Supplementary file 5 — Source Data Fig. 3 [file 44318_2023_14_MOESM5_ESM.zip › Fig 3/C/20220926 rep2 (depicted in the Fig)/FLAGM1 reduced SDS-PAGE.tif]

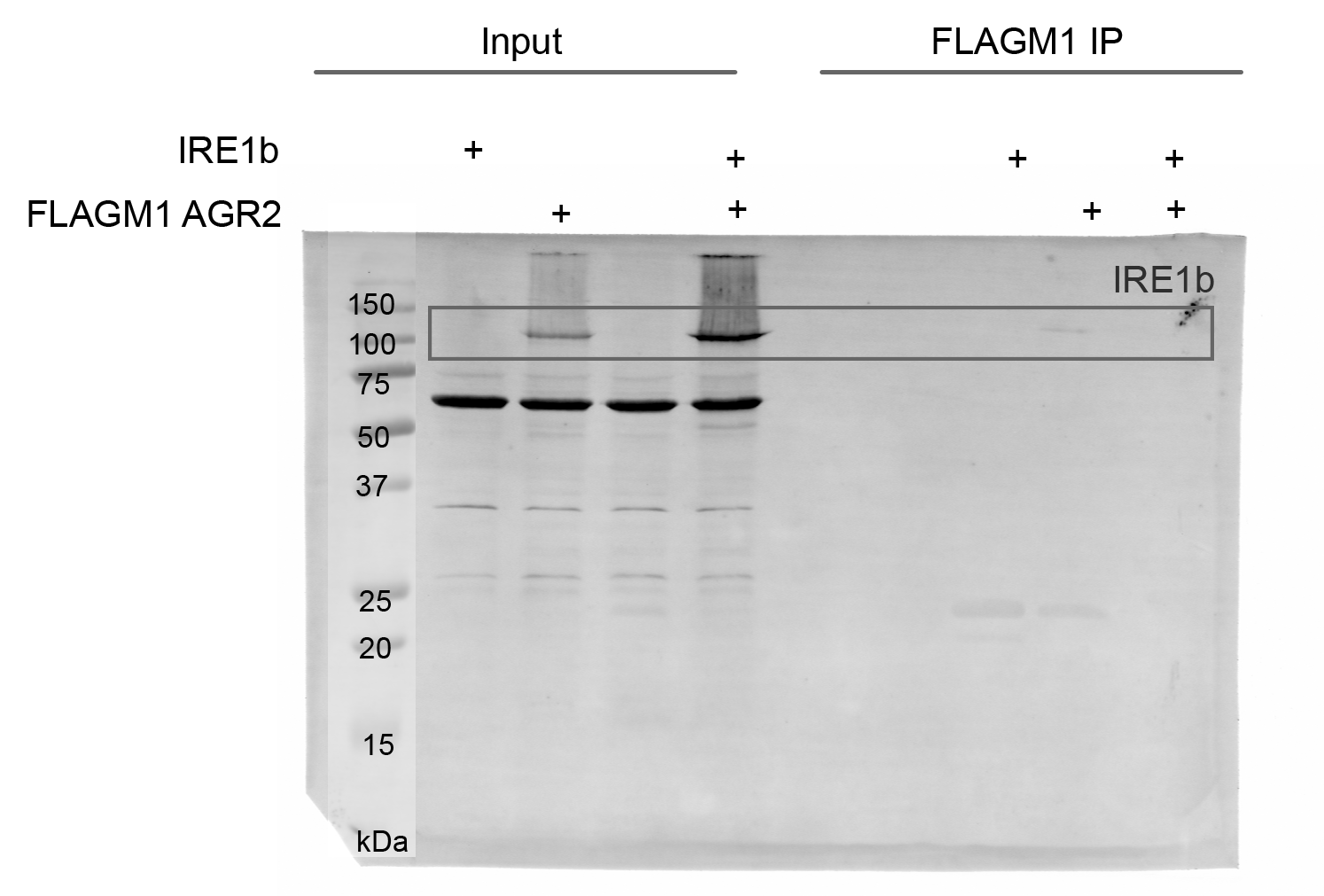

Supplement: Supplementary file 5 — Source Data Fig. 3 [file 44318_2023_14_MOESM5_ESM.zip › Fig 3/C/20220926 rep2 (depicted in the Fig)/NY109 non-reduced SDS-PAGE.tif]

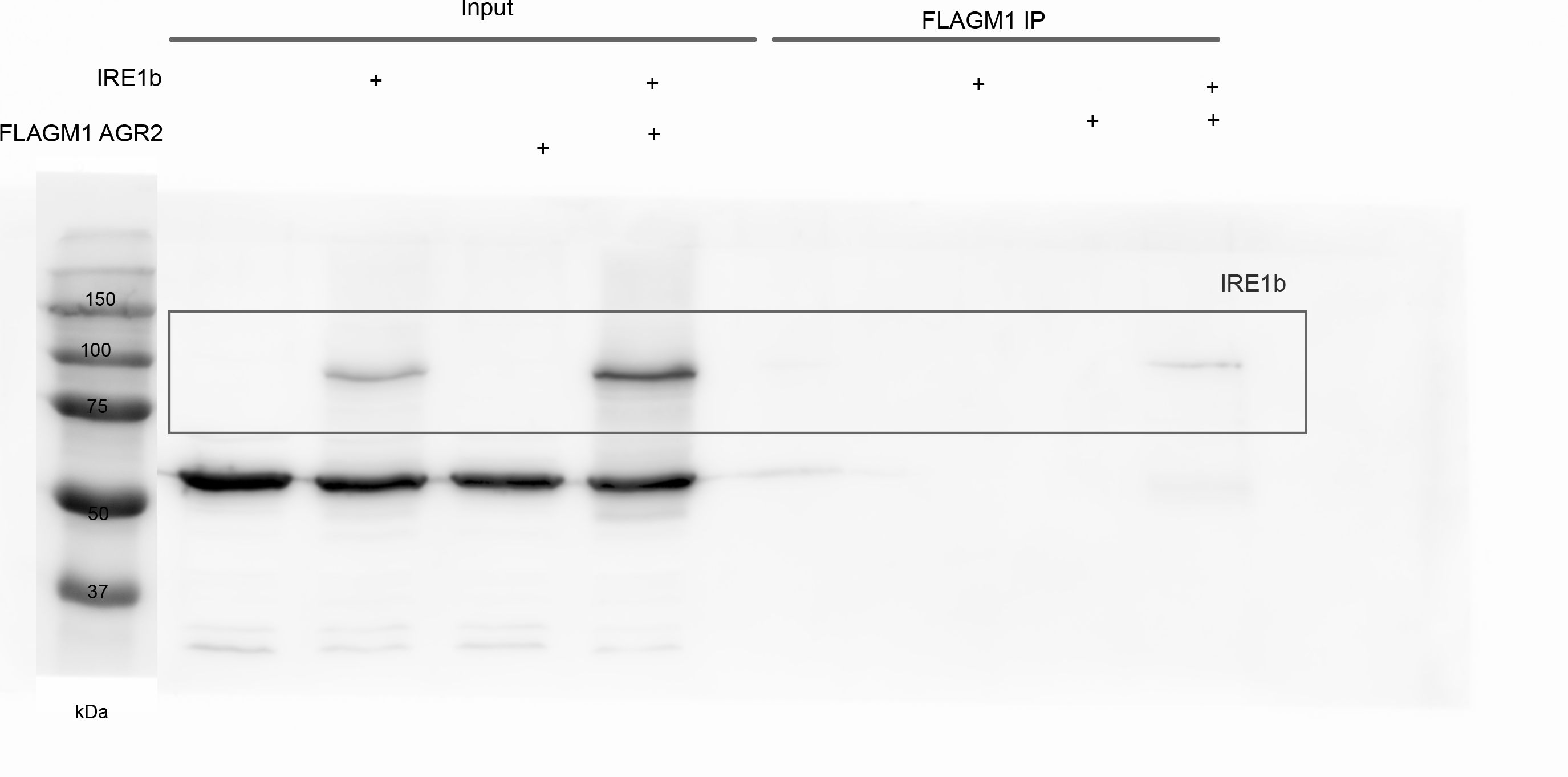

Supplement: Supplementary file 5 — Source Data Fig. 3 [file 44318_2023_14_MOESM5_ESM.zip › Fig 3/C/20220926 rep2 (depicted in the Fig)/NY109 reduced SDS-PAGE.tif]

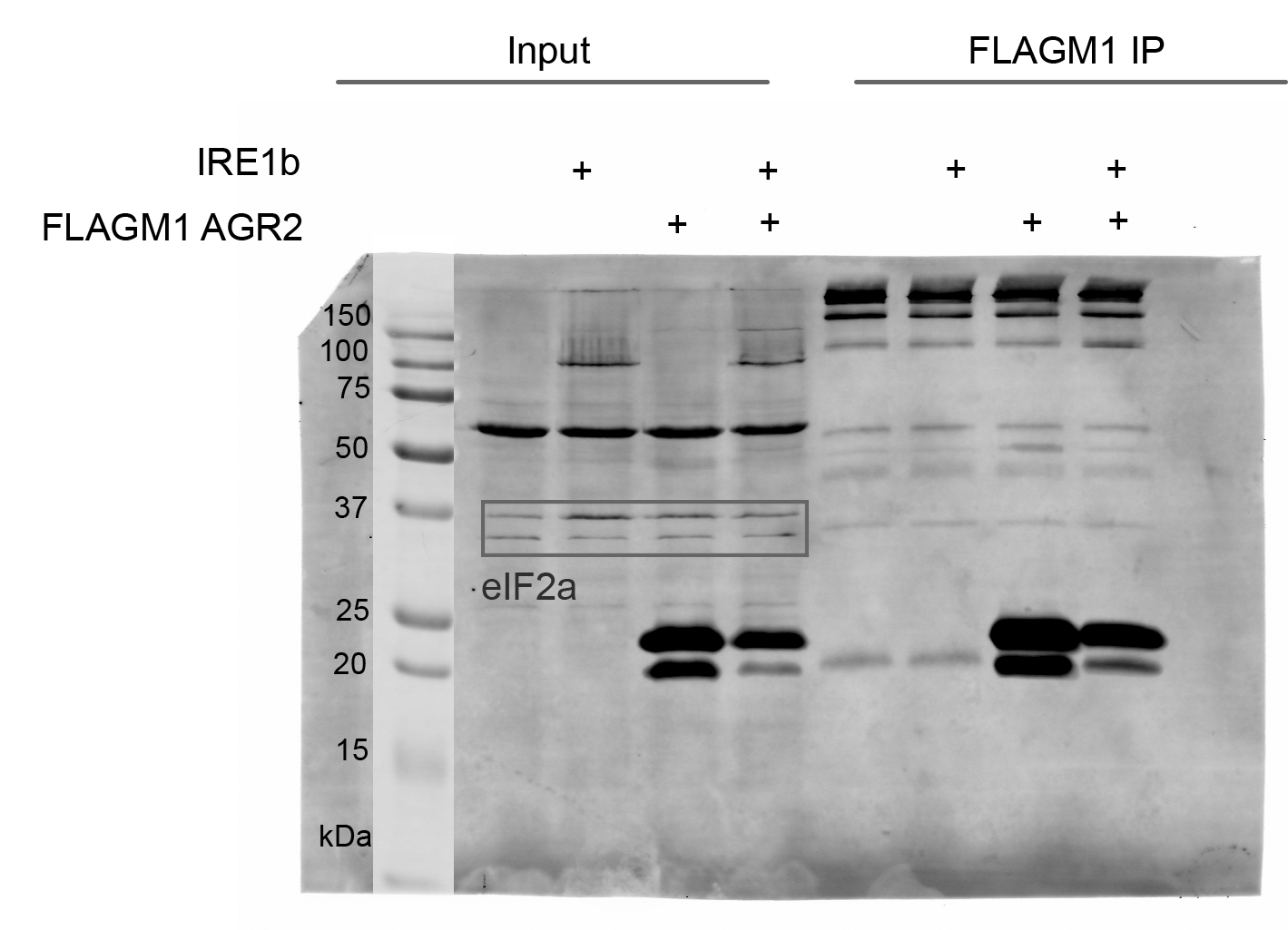

Supplement: Supplementary file 5 — Source Data Fig. 3 [file 44318_2023_14_MOESM5_ESM.zip › Fig 3/C/20221003 rep3/eIF2a non-reduced SDS-PAGE.tif]

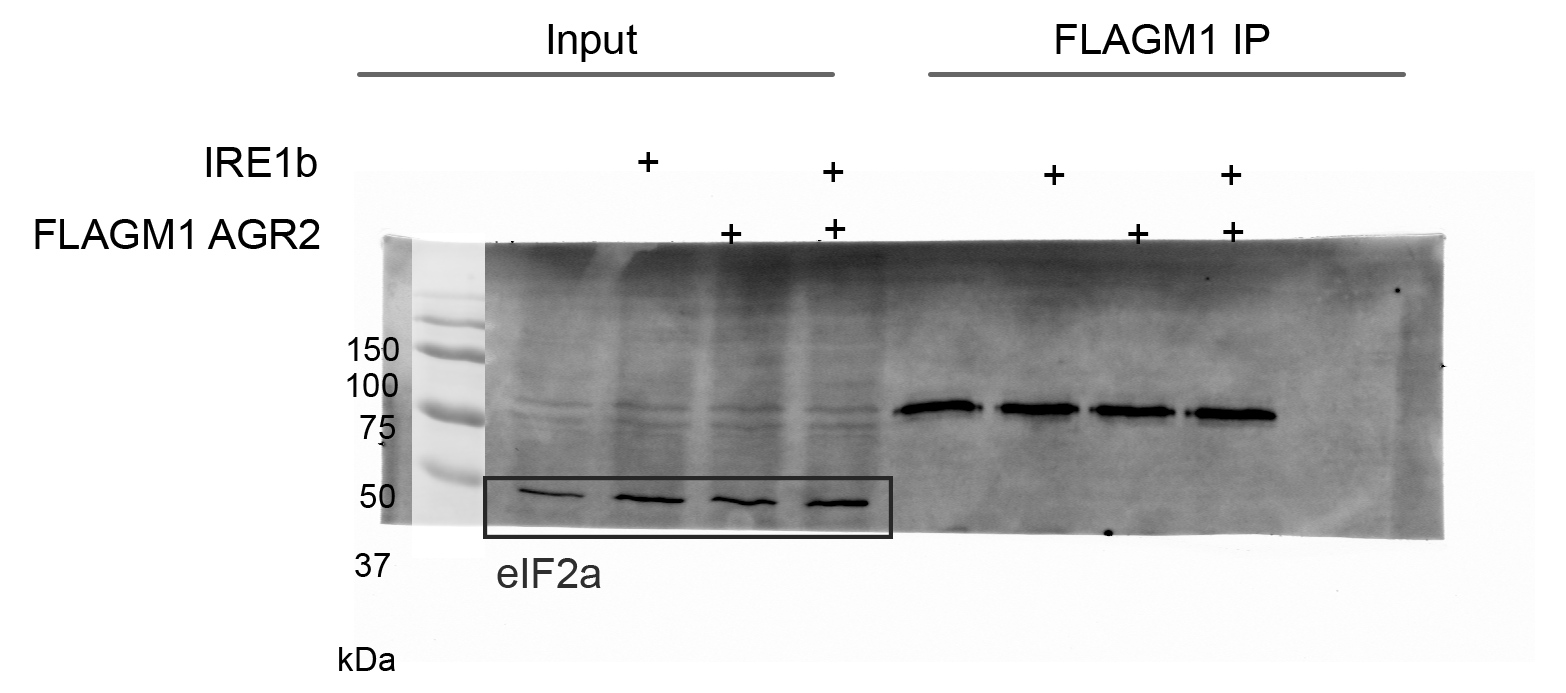

Supplement: Supplementary file 5 — Source Data Fig. 3 [file 44318_2023_14_MOESM5_ESM.zip › Fig 3/C/20221003 rep3/eIF2a reduced SDS-PAGE.tif]

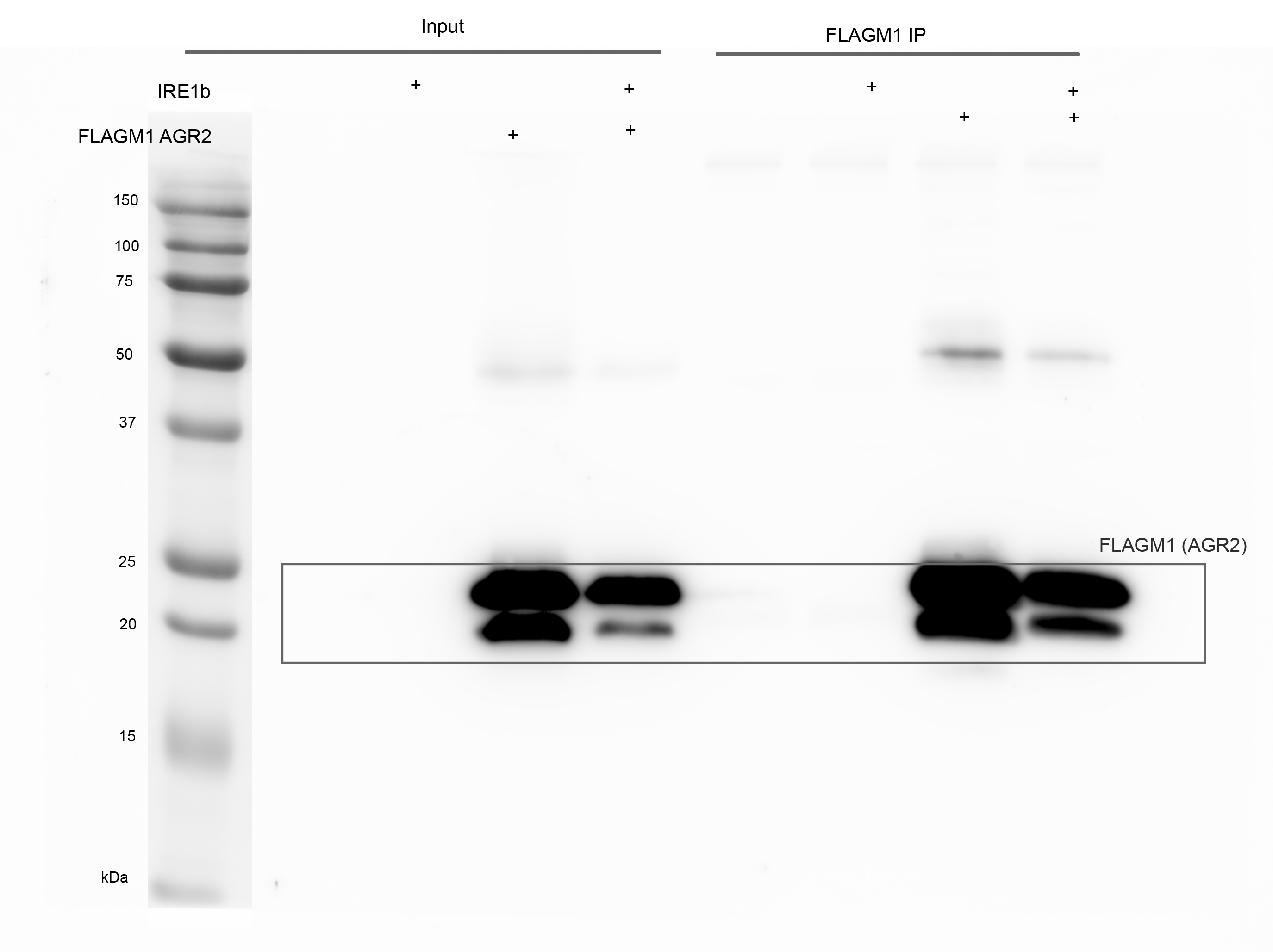

Supplement: Supplementary file 5 — Source Data Fig. 3 [file 44318_2023_14_MOESM5_ESM.zip › Fig 3/C/20221003 rep3/FLAGM1 non-reduced SDS-PAGE.tif]

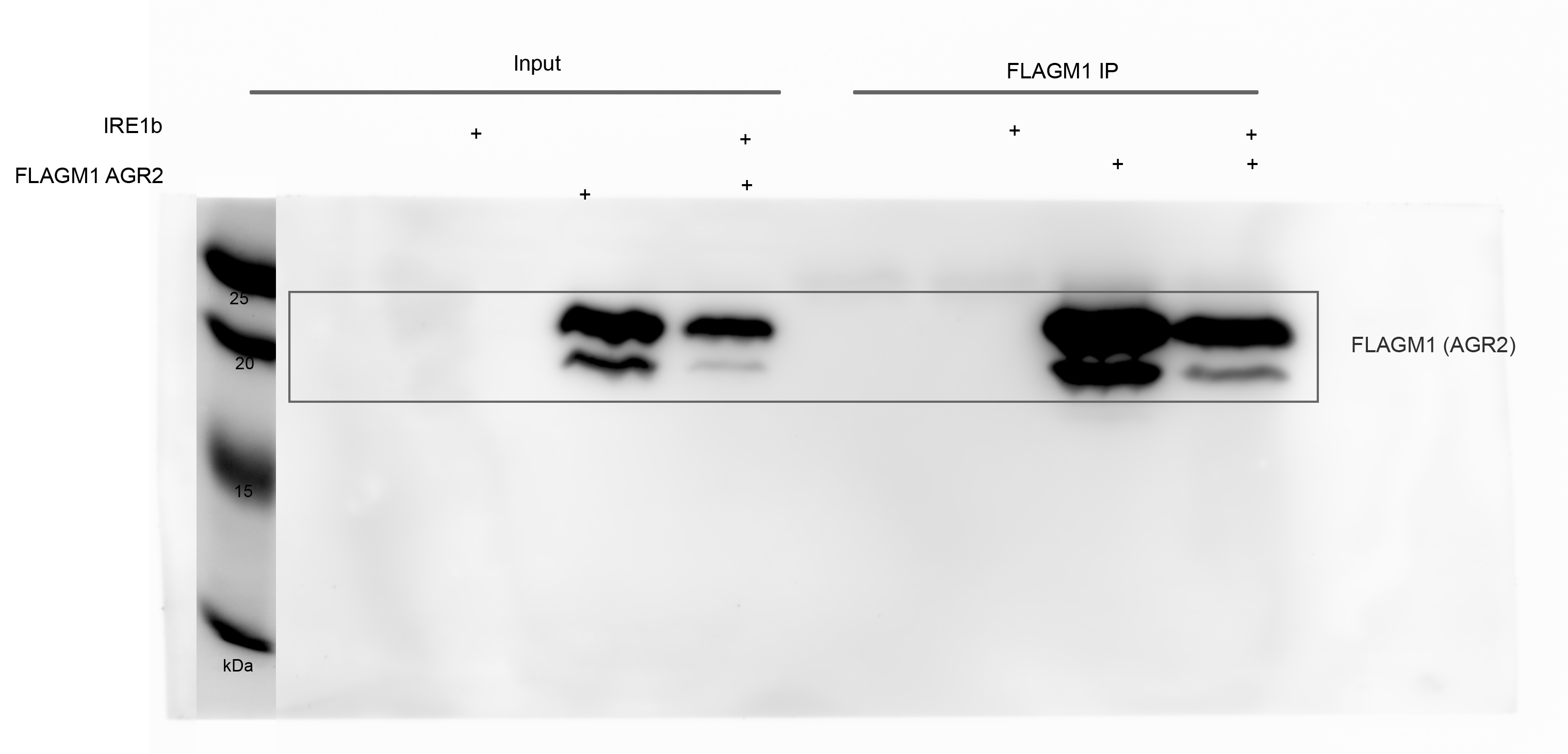

Supplement: Supplementary file 5 — Source Data Fig. 3 [file 44318_2023_14_MOESM5_ESM.zip › Fig 3/C/20221003 rep3/FLAGM1 reduced SDS-PAGE.tif]

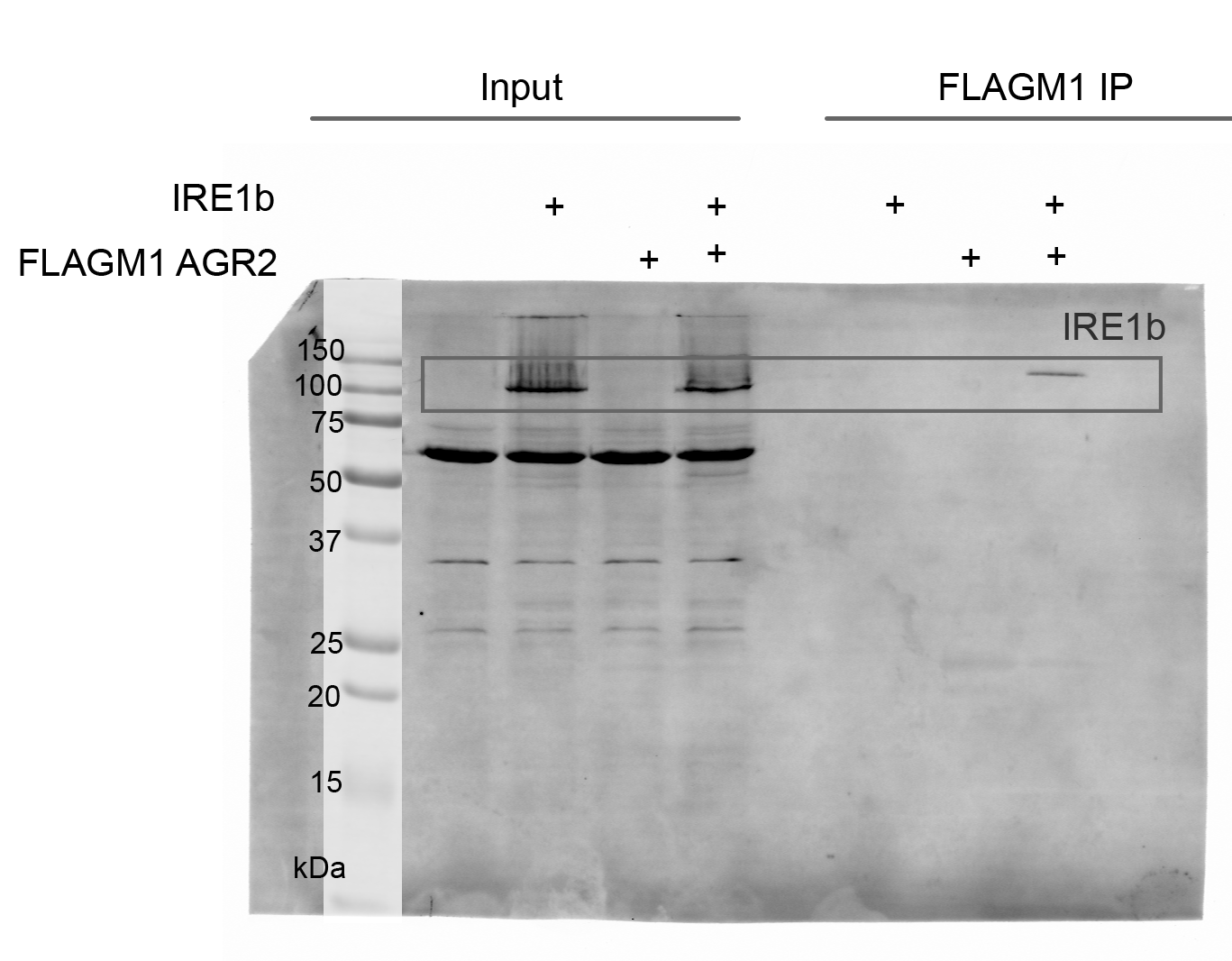

Supplement: Supplementary file 5 — Source Data Fig. 3 [file 44318_2023_14_MOESM5_ESM.zip › Fig 3/C/20221003 rep3/NY109 non-reduced SDS-PAGE.tif]

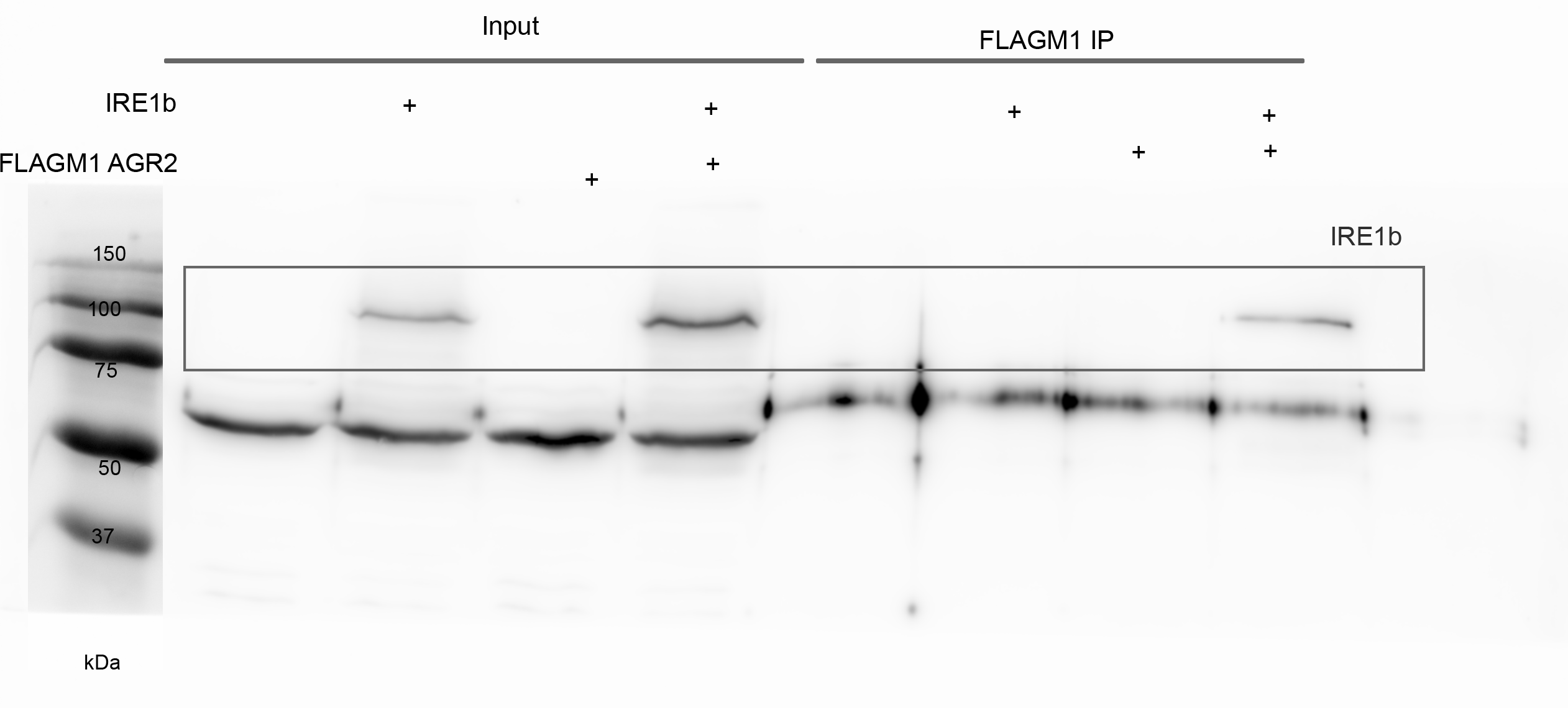

Supplement: Supplementary file 5 — Source Data Fig. 3 [file 44318_2023_14_MOESM5_ESM.zip › Fig 3/C/20221003 rep3/NY109 reduced SDS-PAGE.tif]

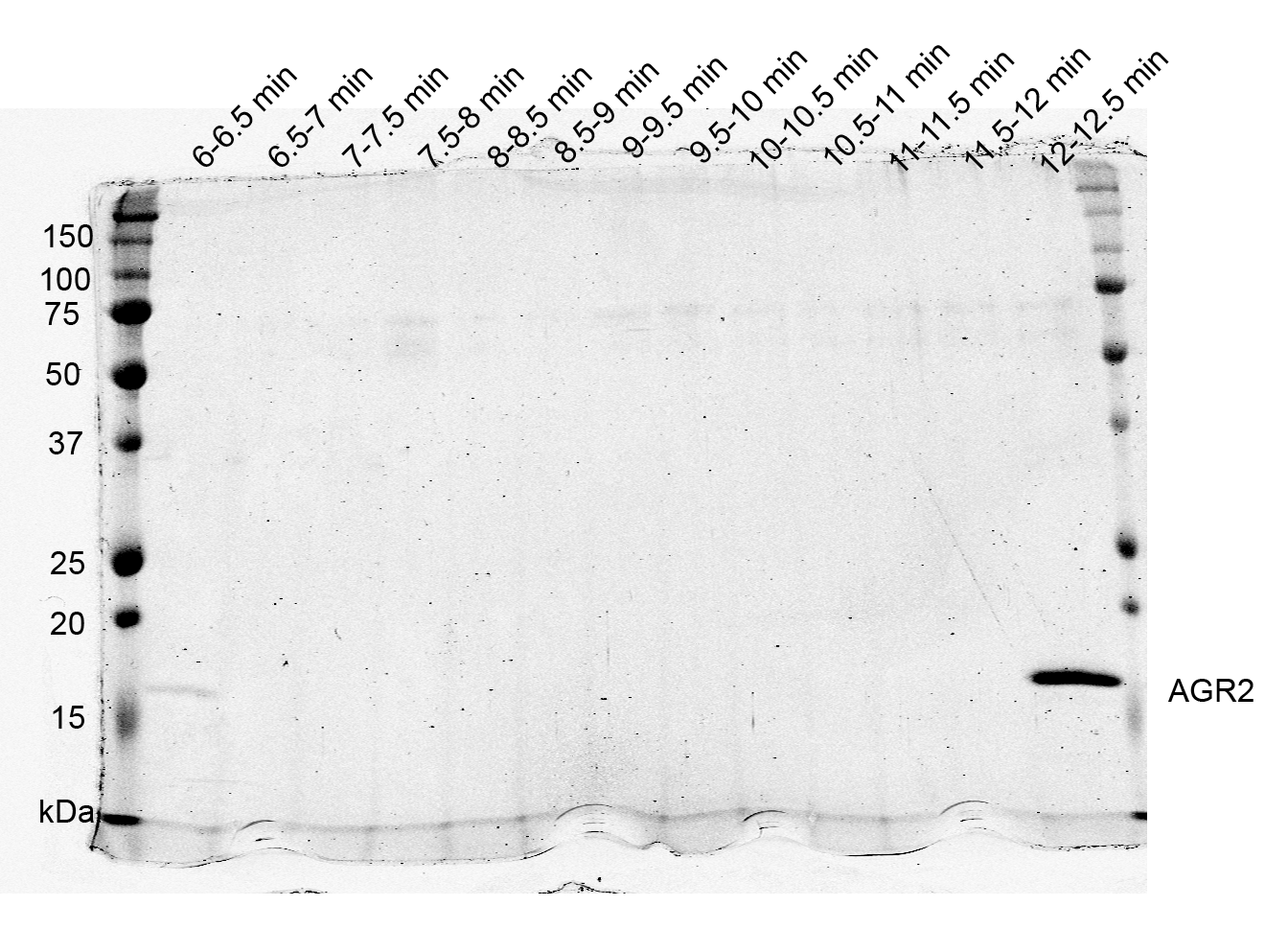

Supplement: Supplementary file 6 — Source Data Fig. 4 [file 44318_2023_14_MOESM6_ESM.zip › Fig 4/A/TCA precipitation/20220210 TCA rep 1/AGR2 alone.tif]

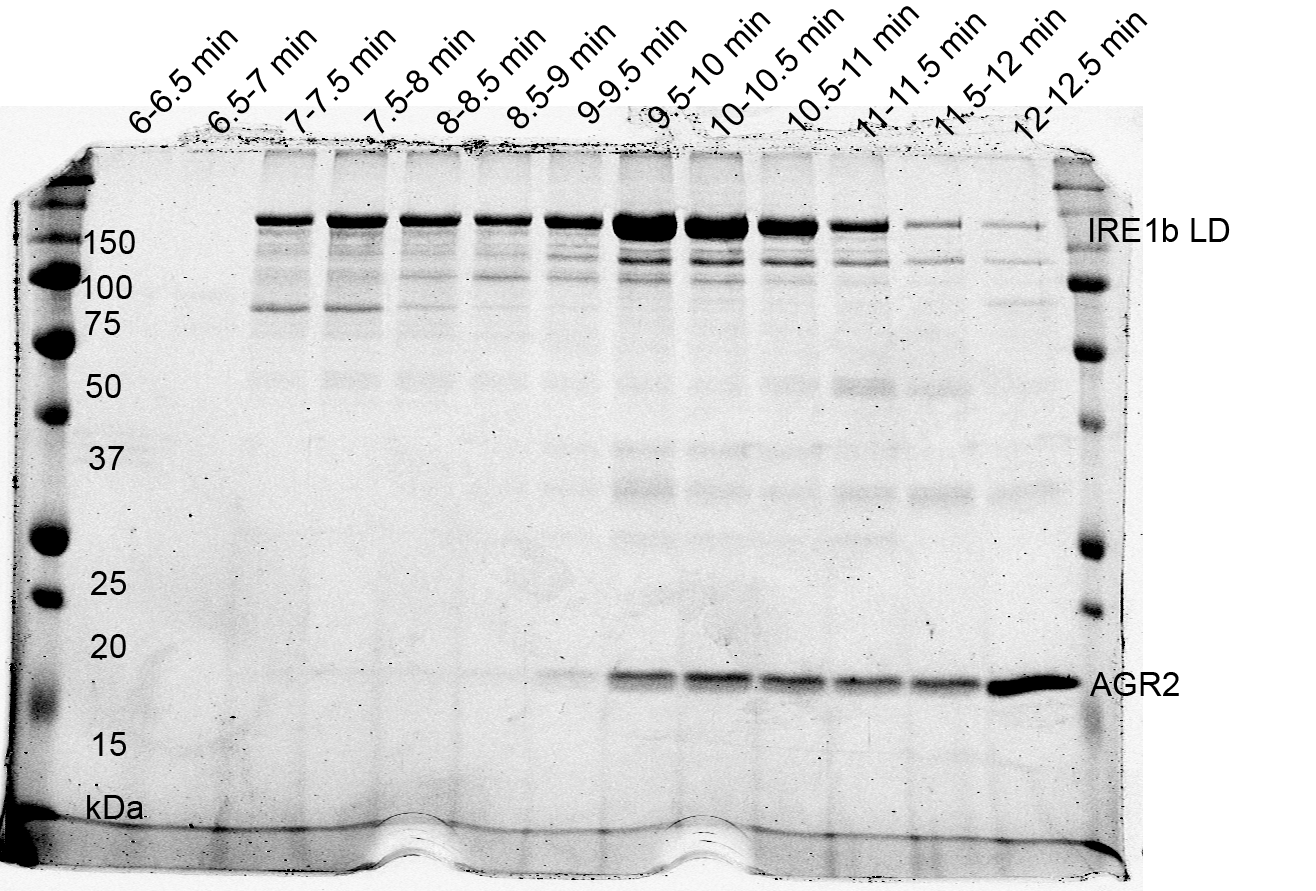

Supplement: Supplementary file 6 — Source Data Fig. 4 [file 44318_2023_14_MOESM6_ESM.zip › Fig 4/A/TCA precipitation/20220210 TCA rep 1/IRE1b + AGR2.tif]

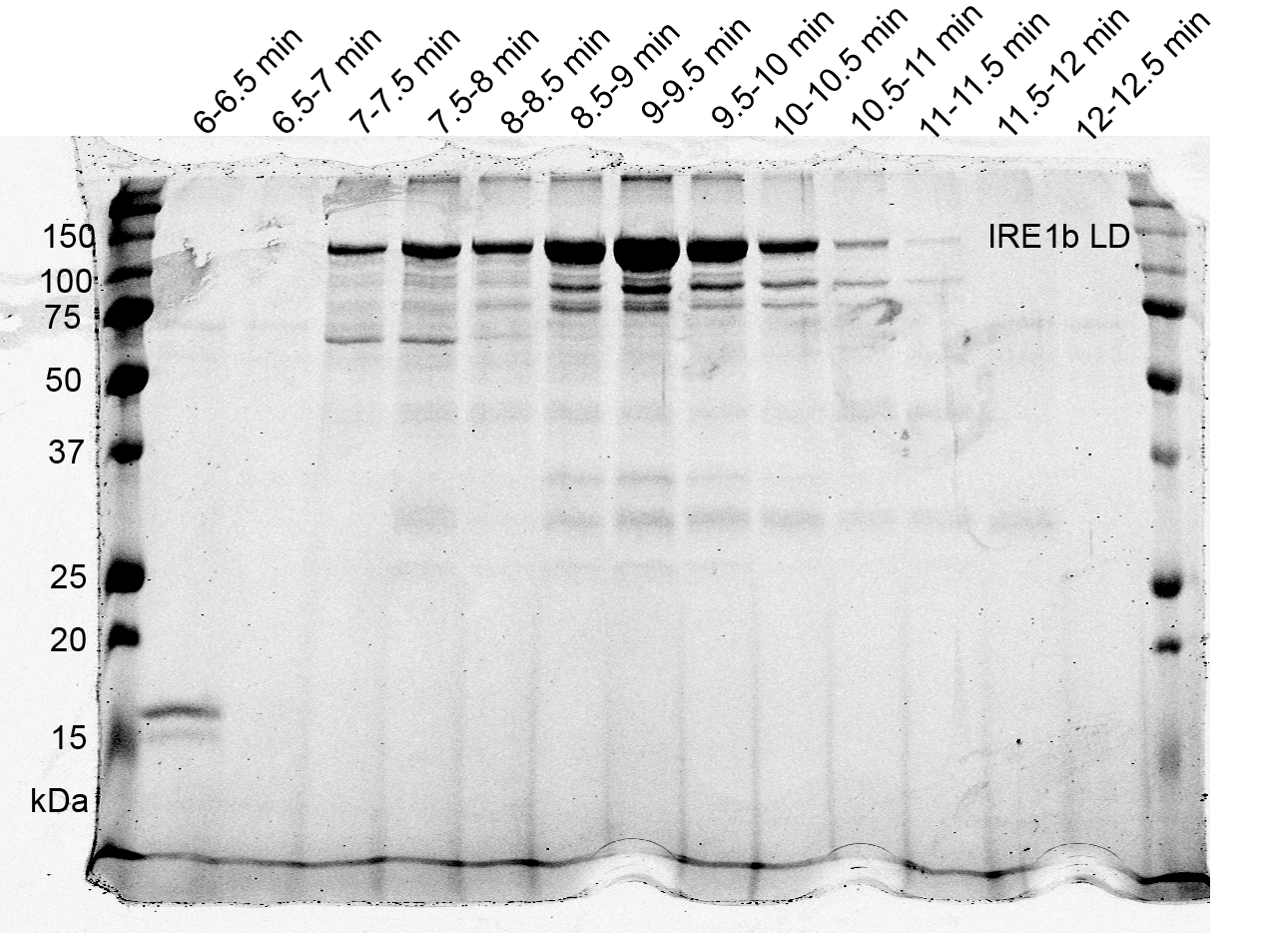

Supplement: Supplementary file 6 — Source Data Fig. 4 [file 44318_2023_14_MOESM6_ESM.zip › Fig 4/A/TCA precipitation/20220210 TCA rep 1/IRE1b alone.tif]

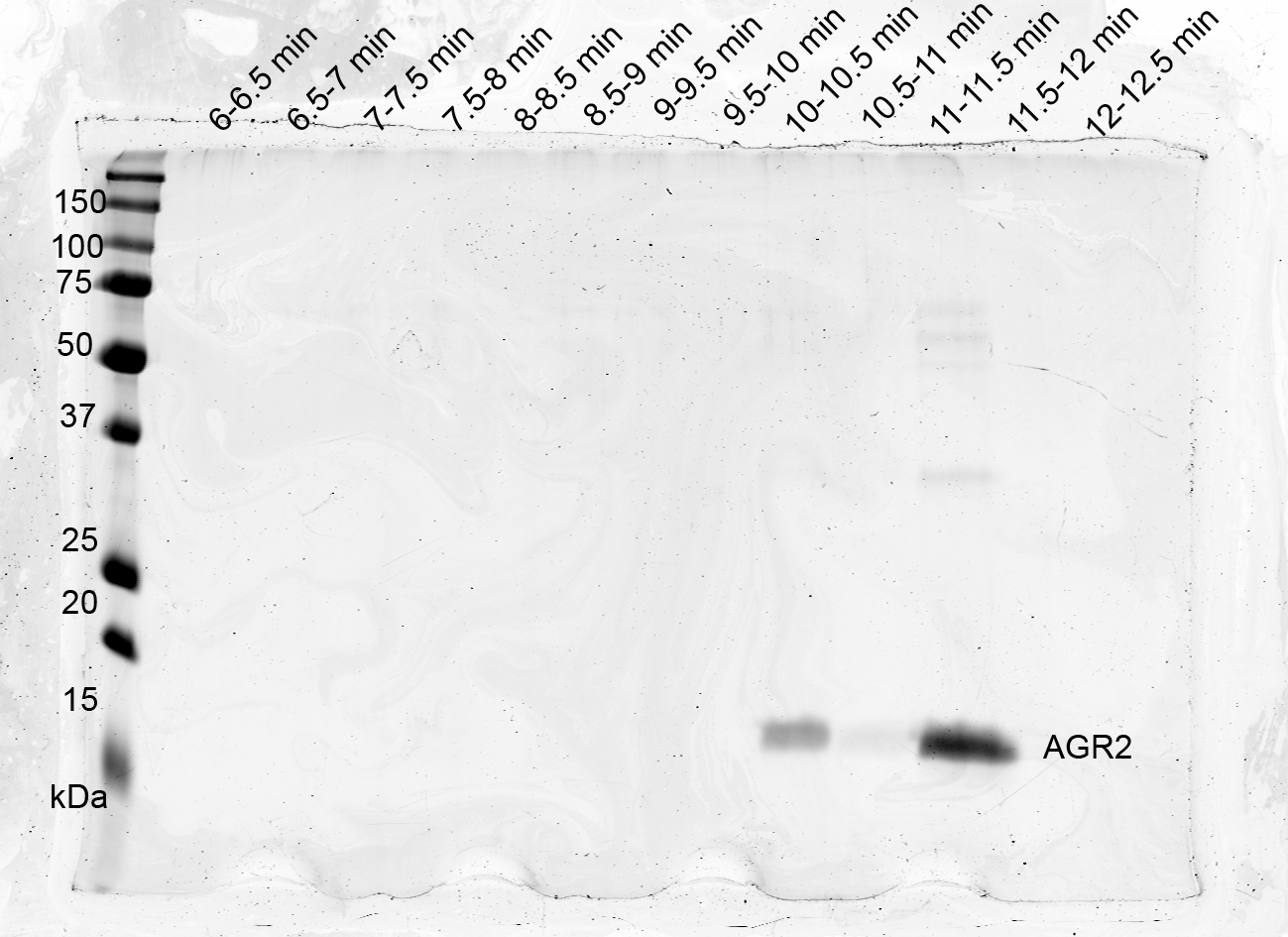

Supplement: Supplementary file 6 — Source Data Fig. 4 [file 44318_2023_14_MOESM6_ESM.zip › Fig 4/A/TCA precipitation/20220519 TCA rep 2/AGR2 alone.tif]

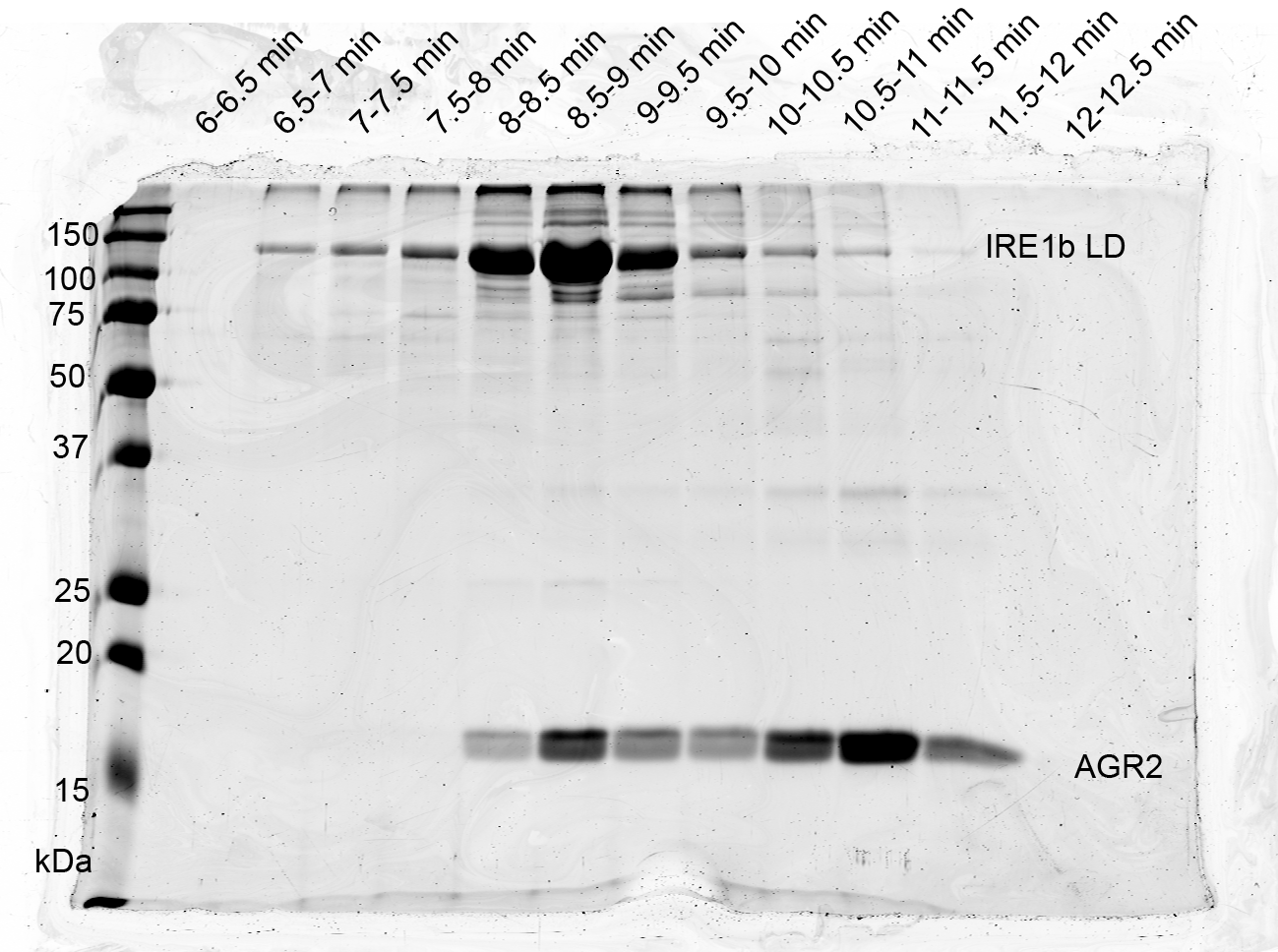

Supplement: Supplementary file 6 — Source Data Fig. 4 [file 44318_2023_14_MOESM6_ESM.zip › Fig 4/A/TCA precipitation/20220519 TCA rep 2/IRE1b + AGR2.tif]

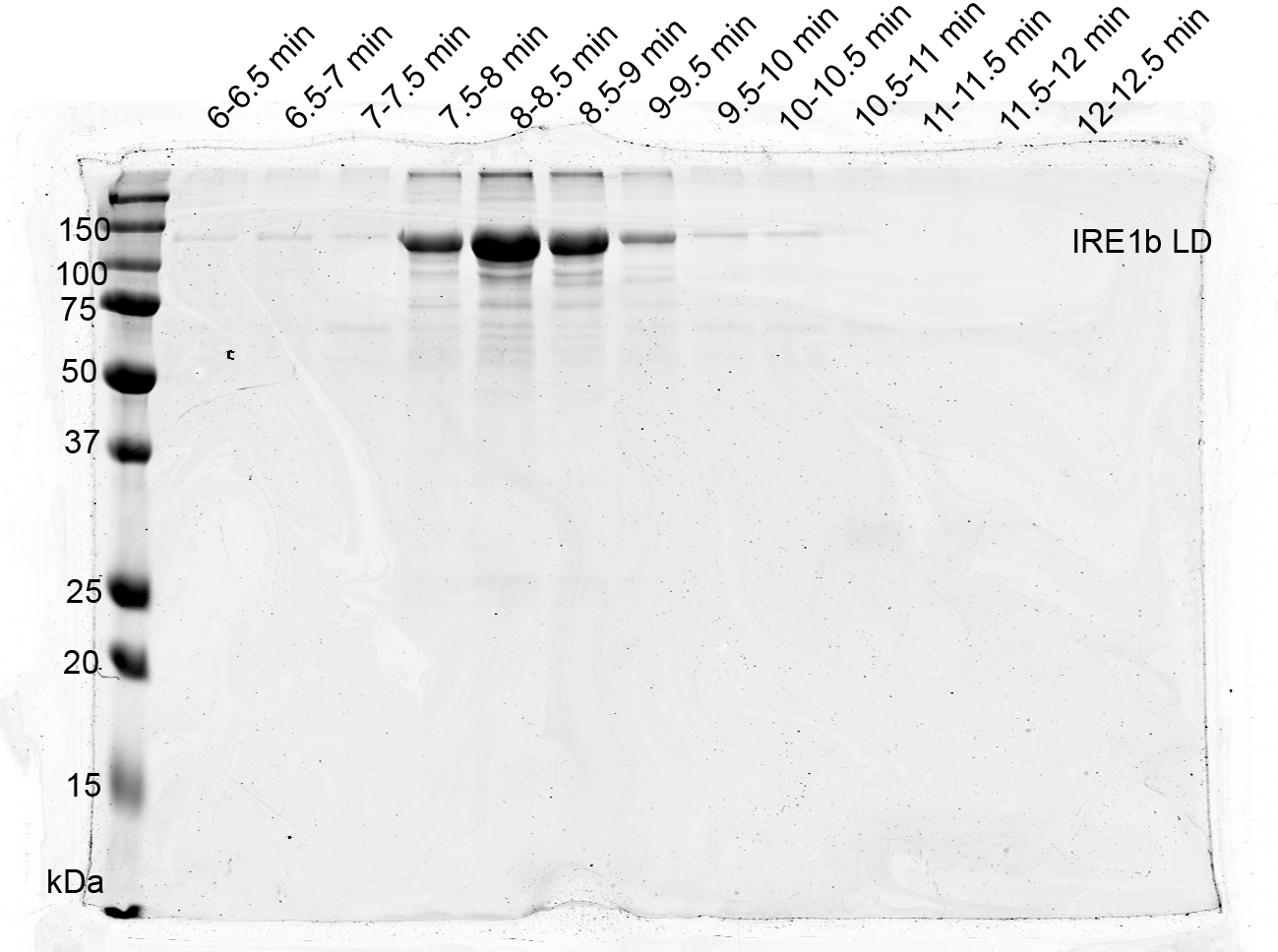

Supplement: Supplementary file 6 — Source Data Fig. 4 [file 44318_2023_14_MOESM6_ESM.zip › Fig 4/A/TCA precipitation/20220519 TCA rep 2/IRE1b alone.tif]

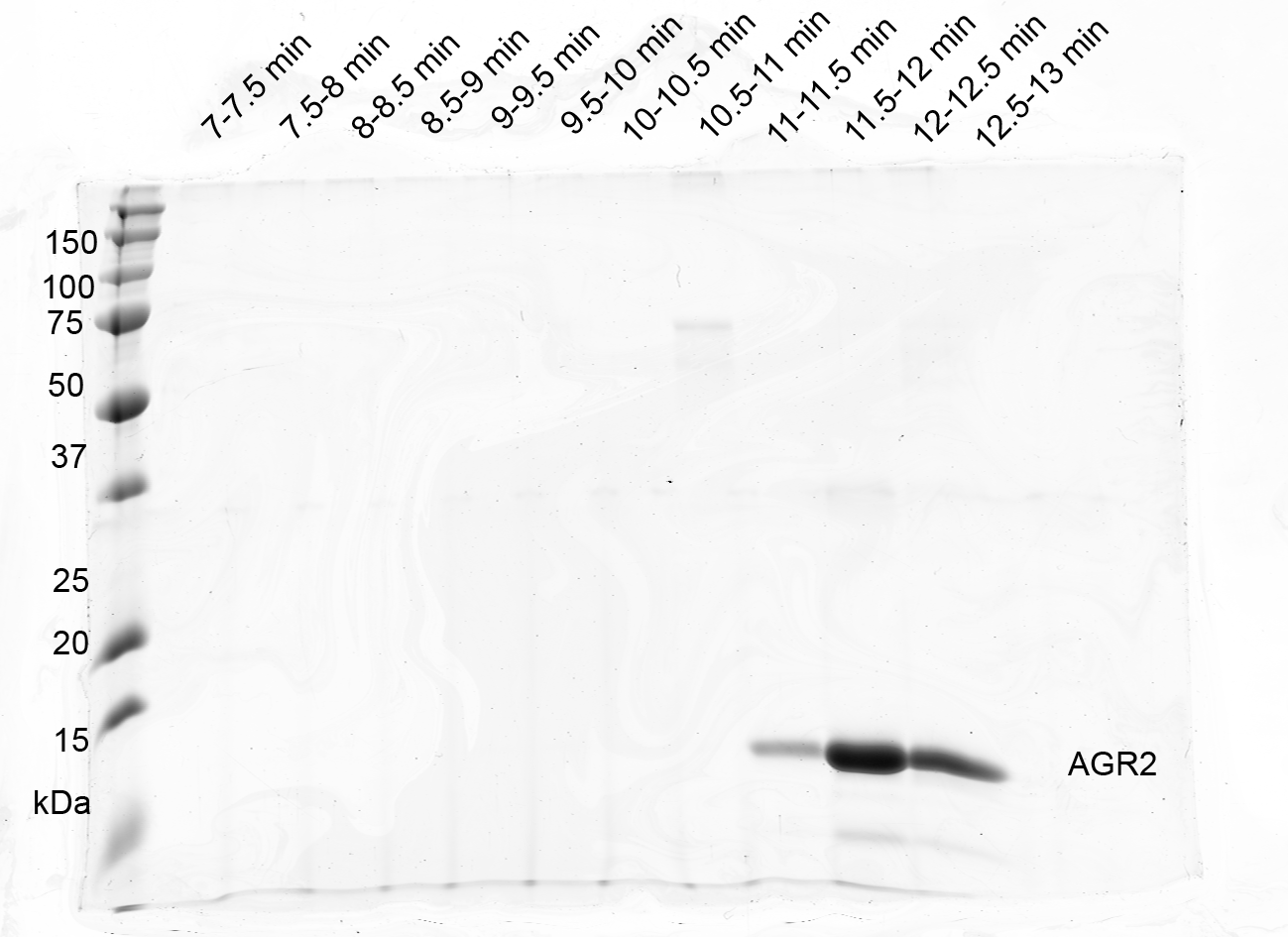

Supplement: Supplementary file 6 — Source Data Fig. 4 [file 44318_2023_14_MOESM6_ESM.zip › Fig 4/A/TCA precipitation/20221007 TCA rep 3 (depicted in Fig)/AGR2 alone.tif]

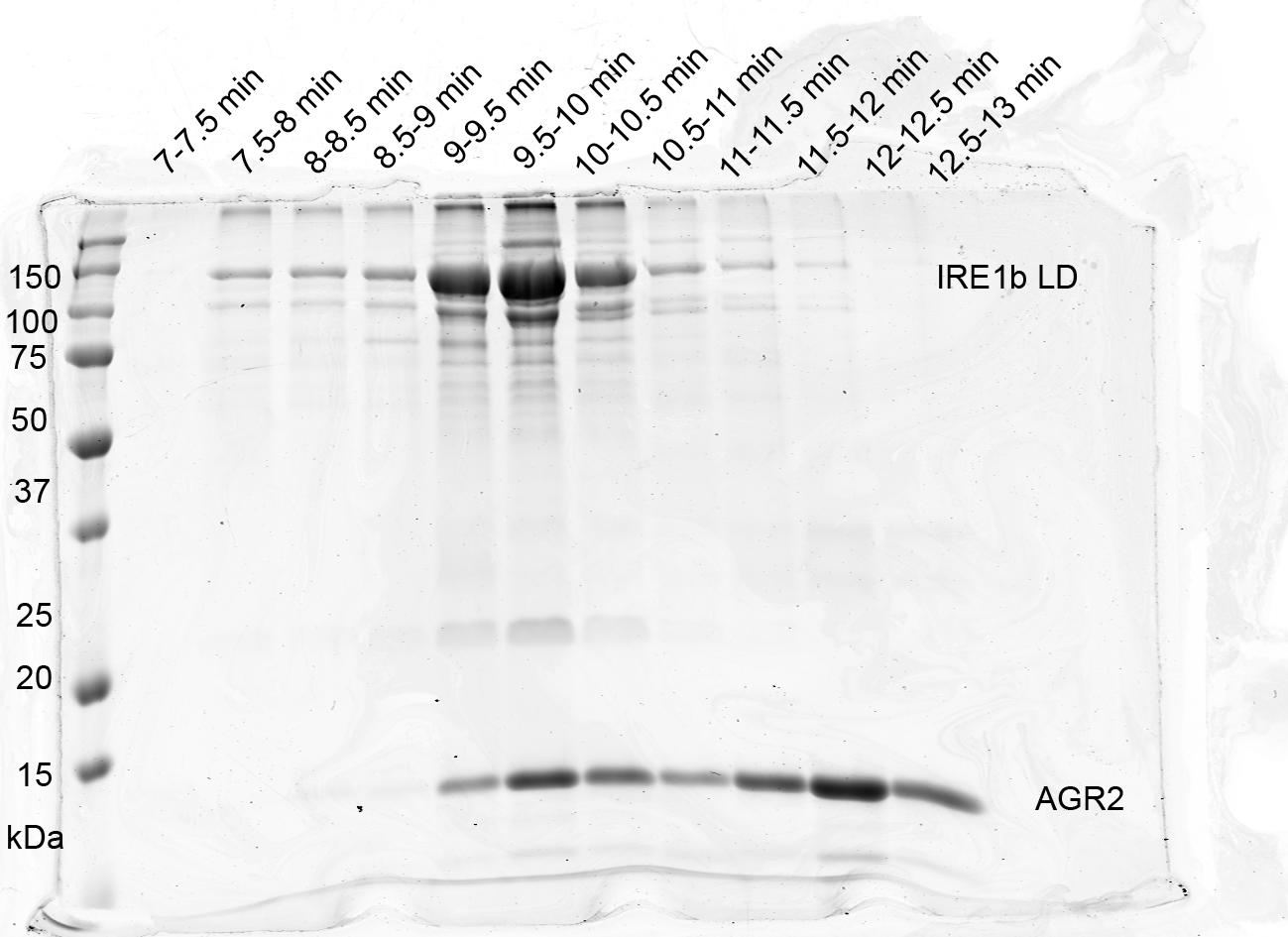

Supplement: Supplementary file 6 — Source Data Fig. 4 [file 44318_2023_14_MOESM6_ESM.zip › Fig 4/A/TCA precipitation/20221007 TCA rep 3 (depicted in Fig)/IRE1b + AGR2.tif]

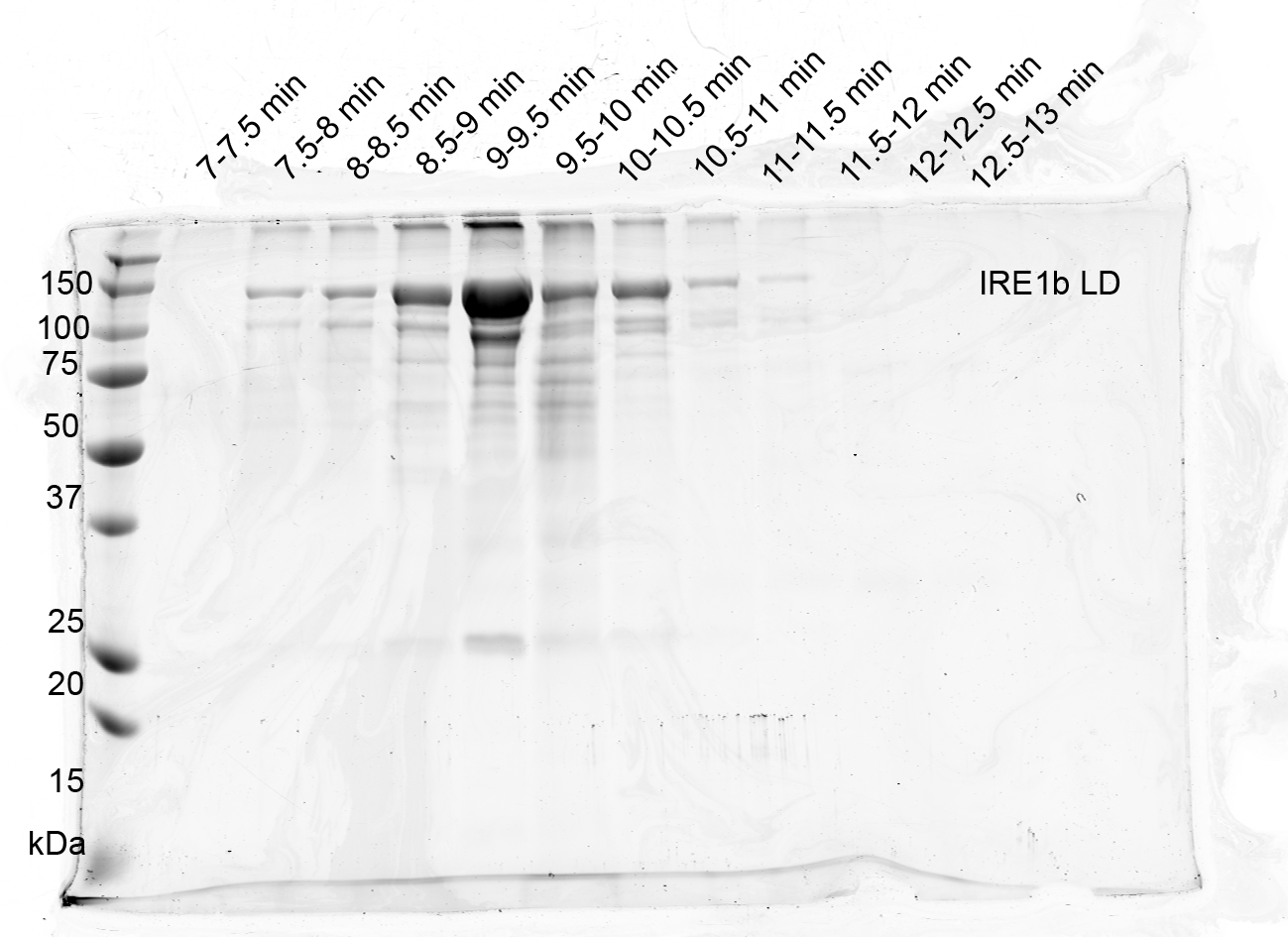

Supplement: Supplementary file 6 — Source Data Fig. 4 [file 44318_2023_14_MOESM6_ESM.zip › Fig 4/A/TCA precipitation/20221007 TCA rep 3 (depicted in Fig)/IRE1b alone.tif]

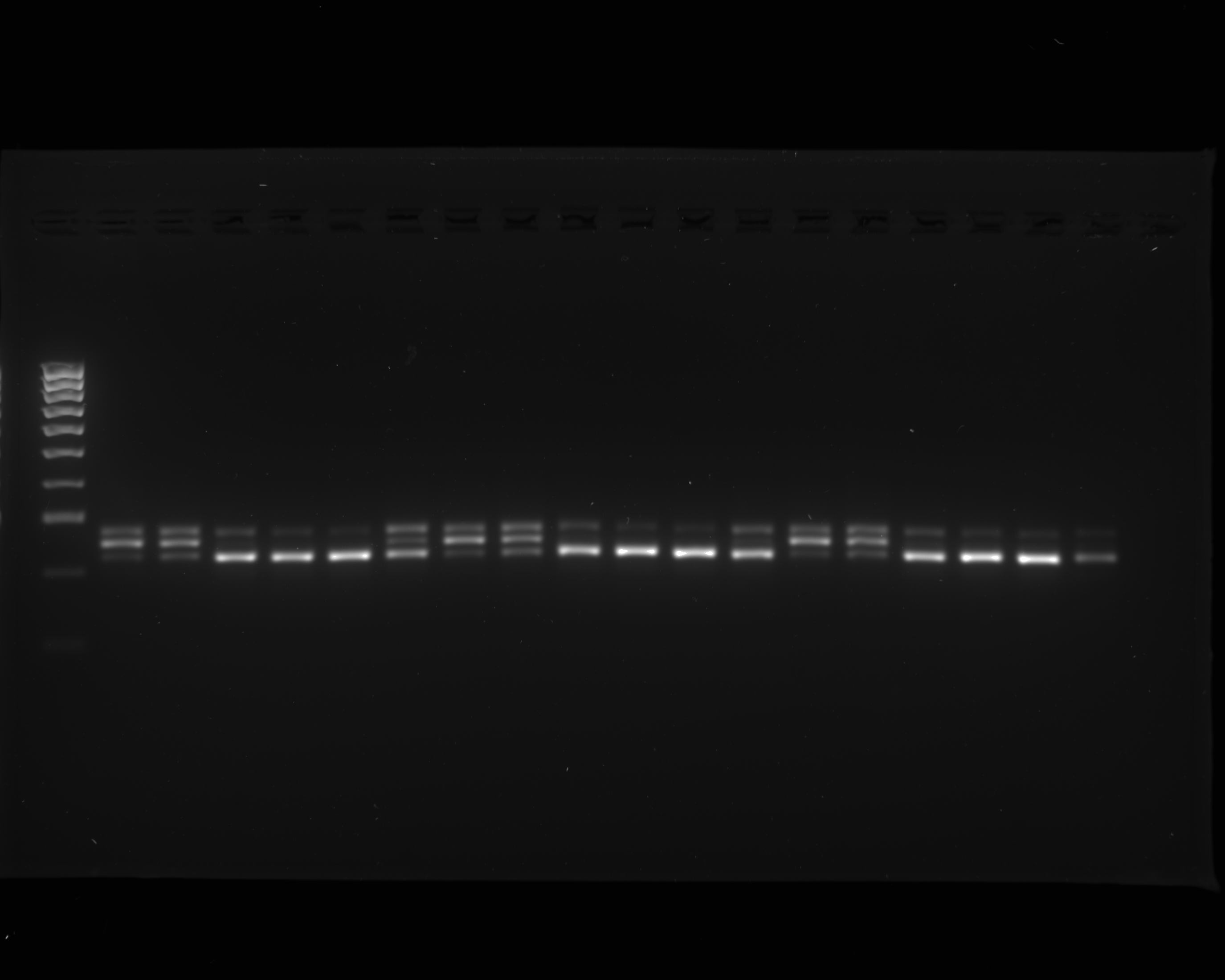

Supplement: Supplementary file 10 — Source Data Fig. 8 [file 44318_2023_14_MOESM10_ESM.zip › Fig 8/B/IRE1a.tif]

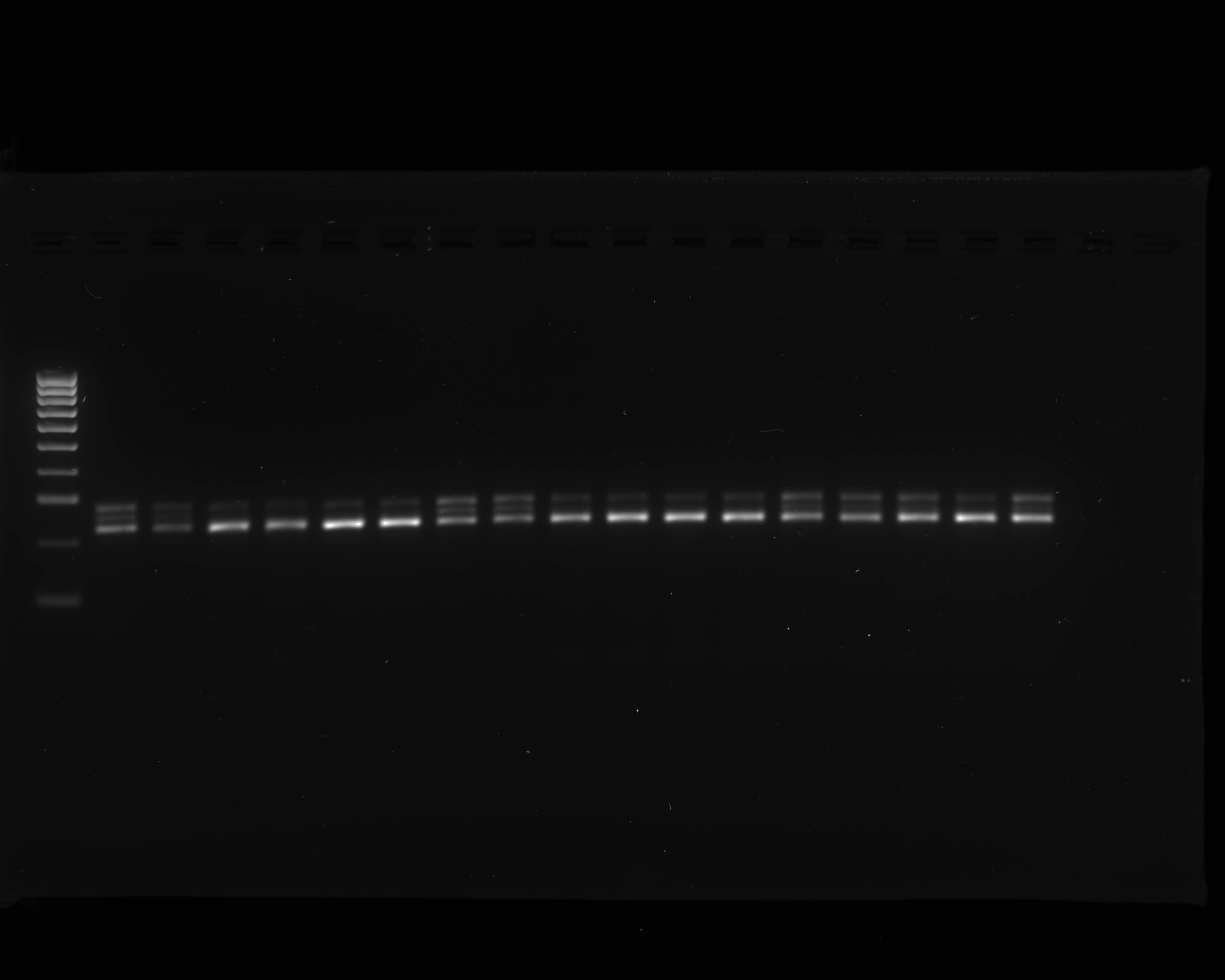

Supplement: Supplementary file 10 — Source Data Fig. 8 [file 44318_2023_14_MOESM10_ESM.zip › Fig 8/B/IRE1aa.tif]

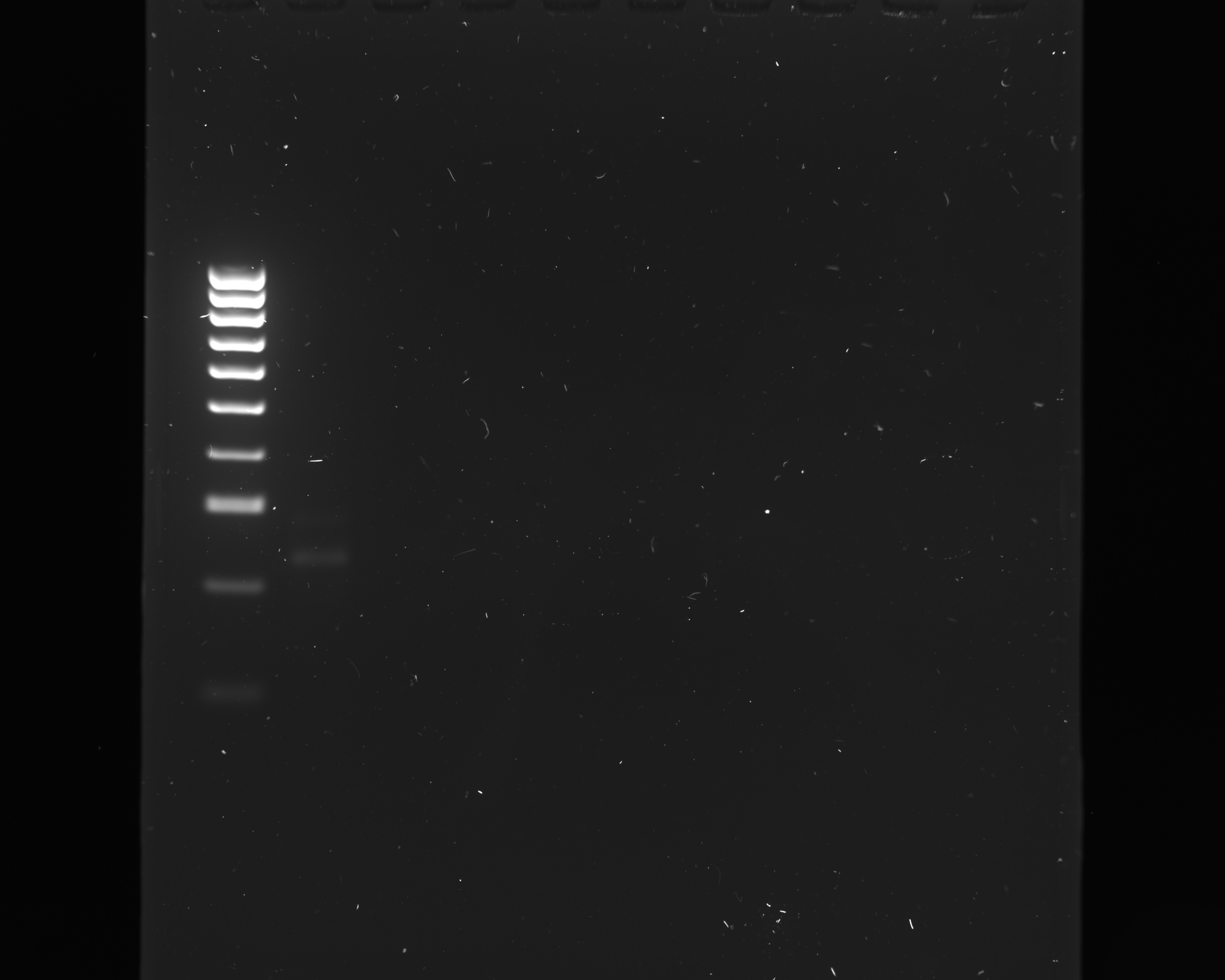

Supplement: Supplementary file 10 — Source Data Fig. 8 [file 44318_2023_14_MOESM10_ESM.zip › Fig 8/B/IRE1aa_repeat.tif]

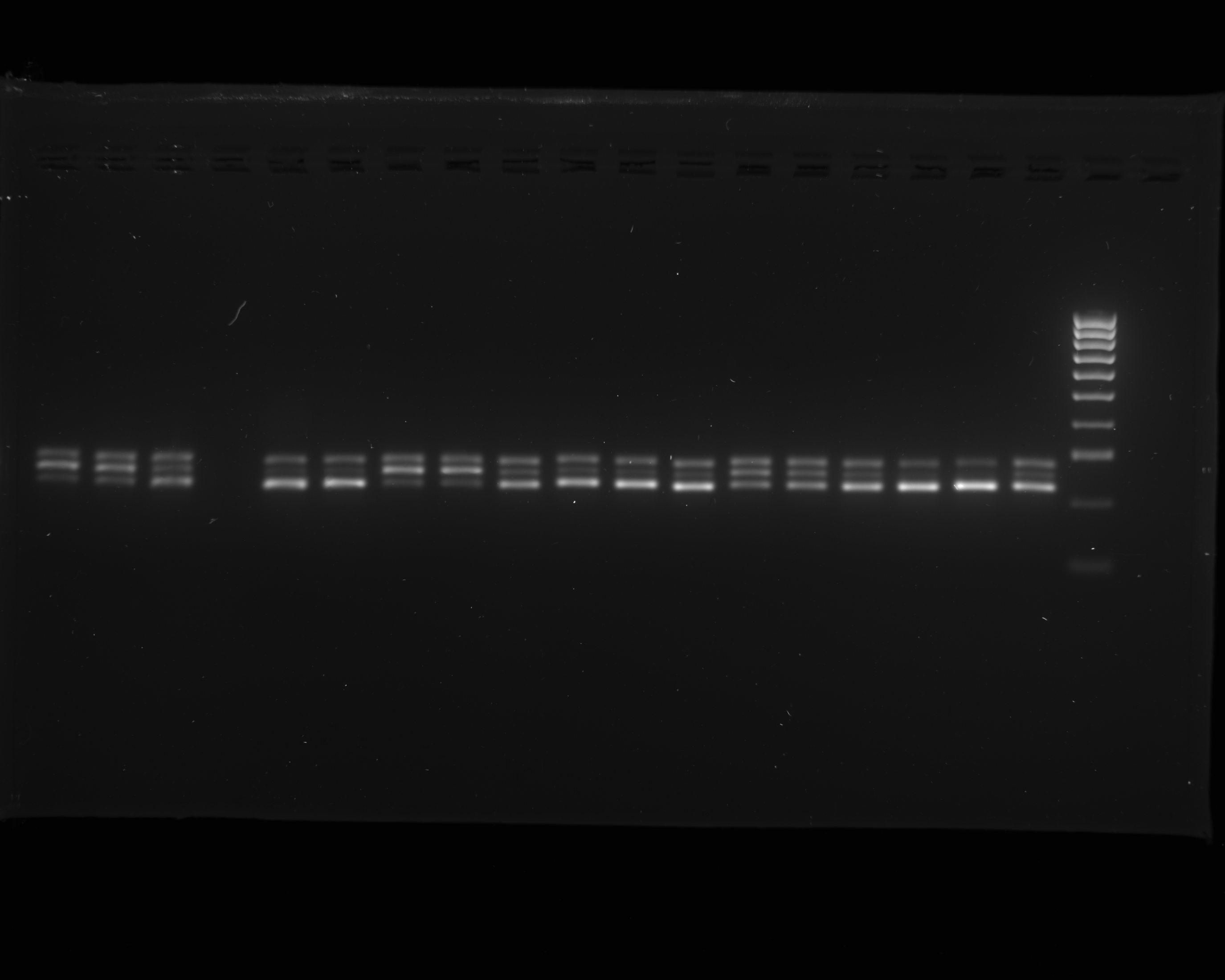

Supplement: Supplementary file 10 — Source Data Fig. 8 [file 44318_2023_14_MOESM10_ESM.zip › Fig 8/B/IRE1ab.tif]

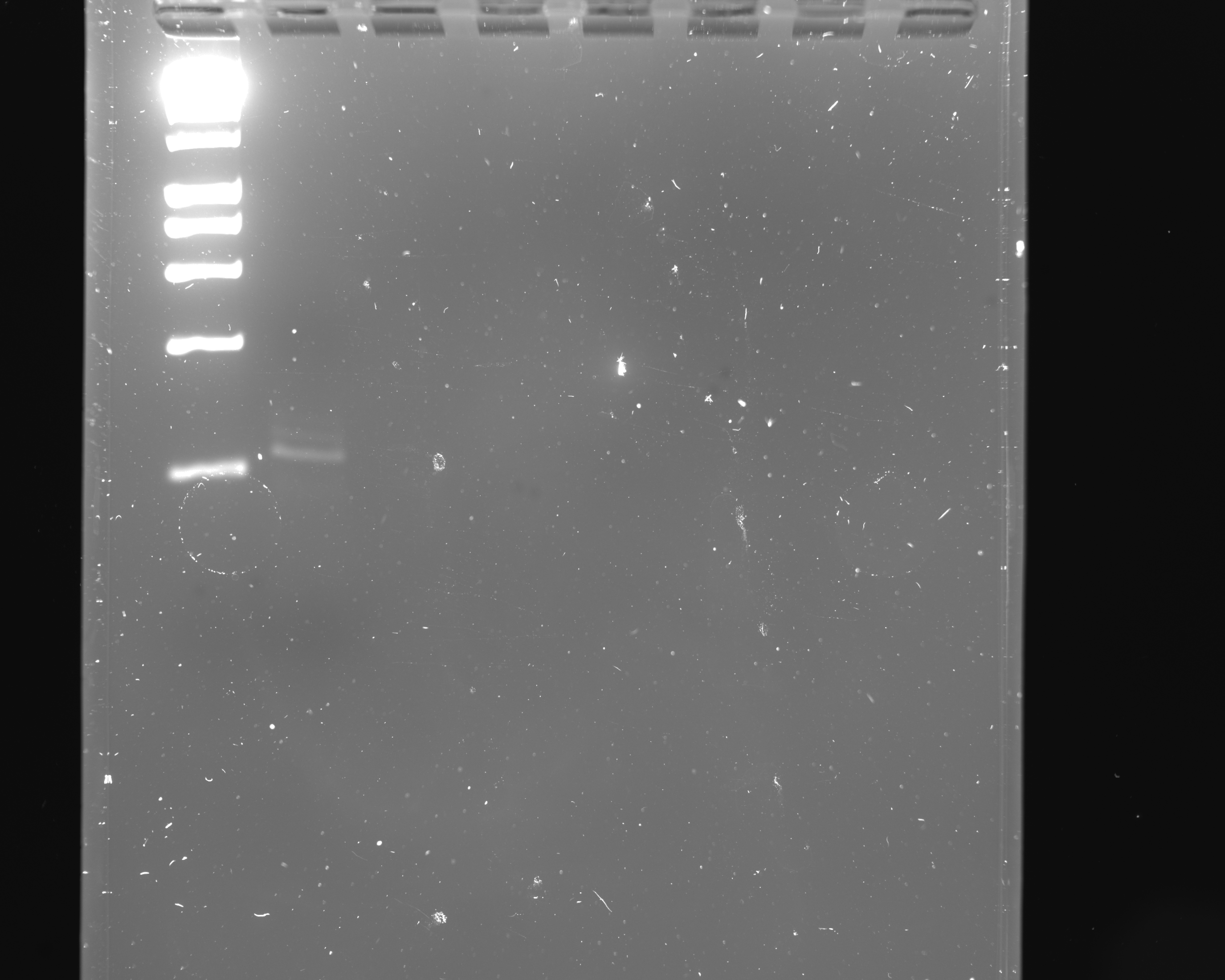

Supplement: Supplementary file 10 — Source Data Fig. 8 [file 44318_2023_14_MOESM10_ESM.zip › Fig 8/B/IRE1ab_repeat.tif]

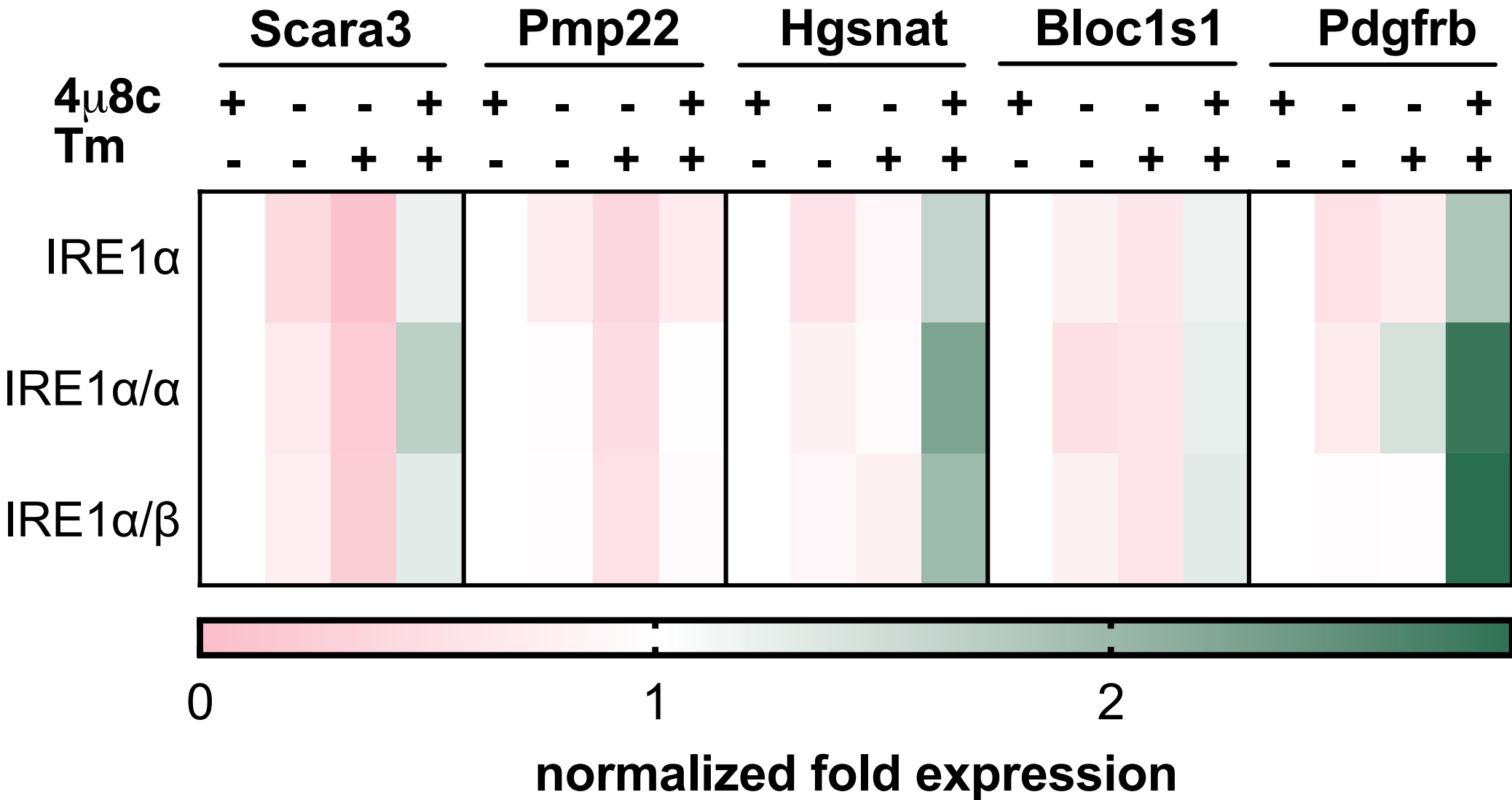

Supplement: Supplementary file 10 — Source Data Fig. 8 [file 44318_2023_14_MOESM10_ESM.zip › Fig 8/D/RIDD target 4u8c heat map analysis.pdf]
