# Supplementary material for: The IRE1β-mediated unfolded protein response is repressed by the chaperone AGR2 in mucin producing cells
Source: EMBO J. 2023 Dec 18;43(5):719–53. doi: 10.1038/s44318-023-00014-z (PMC10907699; doi:10.1038/s44318-023-00014-z)
Supplement: Supplementary file 10 — Source Data Fig. 8 [file 44318_2023_14_MOESM10_ESM.zip › Fig 8/B/Gel annotation.pptx]

## Slide 1
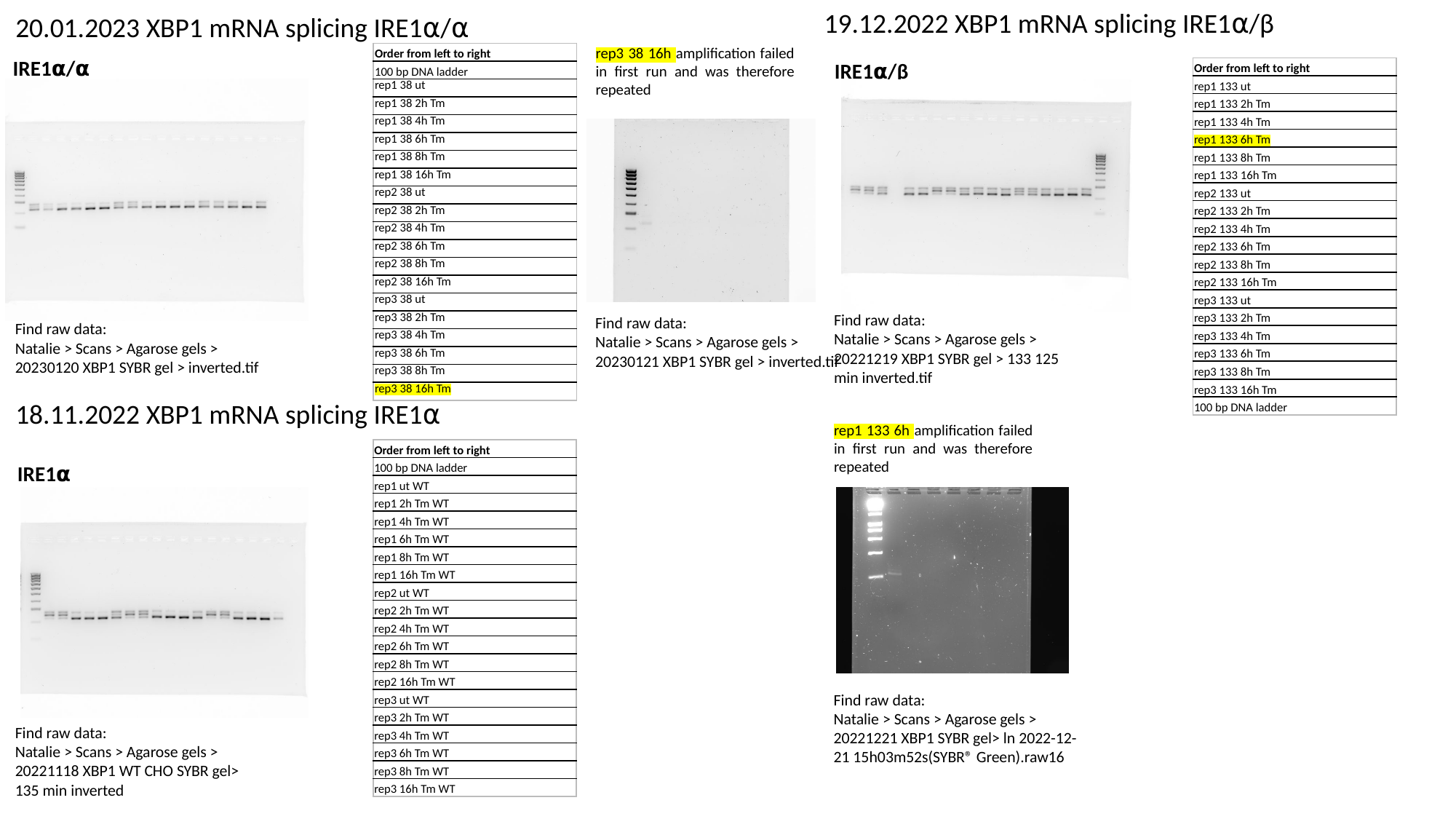

19.12.2022 XBP1 mRNA splicing IRE1⍺/β
20.01.2023 XBP1 mRNA splicing IRE1⍺/⍺
rep3 38 16h amplification failed in first run and was therefore repeated
| Order from left to right |
| --- |
| 100 bp DNA ladder |
| rep1 38 ut |
| rep1 38 2h Tm |
| rep1 38 4h Tm |
| rep1 38 6h Tm |
| rep1 38 8h Tm |
| rep1 38 16h Tm |
| rep2 38 ut |
| rep2 38 2h Tm |
| rep2 38 4h Tm |
| rep2 38 6h Tm |
| rep2 38 8h Tm |
| rep2 38 16h Tm |
| rep3 38 ut |
| rep3 38 2h Tm |
| rep3 38 4h Tm |
| rep3 38 6h Tm |
| rep3 38 8h Tm |
| rep3 38 16h Tm |
IRE1⍺/⍺
IRE1⍺/β
| Order from left to right |
| --- |
| rep1 133 ut |
| rep1 133 2h Tm |
| rep1 133 4h Tm |
| rep1 133 6h Tm |
| rep1 133 8h Tm |
| rep1 133 16h Tm |
| rep2 133 ut |
| rep2 133 2h Tm |
| rep2 133 4h Tm |
| rep2 133 6h Tm |
| rep2 133 8h Tm |
| rep2 133 16h Tm |
| rep3 133 ut |
| rep3 133 2h Tm |
| rep3 133 4h Tm |
| rep3 133 6h Tm |
| rep3 133 8h Tm |
| rep3 133 16h Tm |
| 100 bp DNA ladder |
Find raw data:
Natalie > Scans > Agarose gels > 20221219 XBP1 SYBR gel > 133 125 min inverted.tif
Find raw data:
Natalie > Scans > Agarose gels > 20230121 XBP1 SYBR gel > inverted.tif
Find raw data:
Natalie > Scans > Agarose gels > 20230120 XBP1 SYBR gel > inverted.tif
18.11.2022 XBP1 mRNA splicing IRE1⍺
rep1 133 6h amplification failed in first run and was therefore repeated
| Order from left to right |
| --- |
| 100 bp DNA ladder |
| rep1 ut WT |
| rep1 2h Tm WT |
| rep1 4h Tm WT |
| rep1 6h Tm WT |
| rep1 8h Tm WT |
| rep1 16h Tm WT |
| rep2 ut WT |
| rep2 2h Tm WT |
| rep2 4h Tm WT |
| rep2 6h Tm WT |
| rep2 8h Tm WT |
| rep2 16h Tm WT |
| rep3 ut WT |
| rep3 2h Tm WT |
| rep3 4h Tm WT |
| rep3 6h Tm WT |
| rep3 8h Tm WT |
| rep3 16h Tm WT |
IRE1⍺
Find raw data:
Natalie > Scans > Agarose gels > 20221221 XBP1 SYBR gel> ln 2022-12-21 15h03m52s(SYBR® Green).raw16
Find raw data:
Natalie > Scans > Agarose gels > 20221118 XBP1 WT CHO SYBR gel> 135 min inverted

## Slide 2
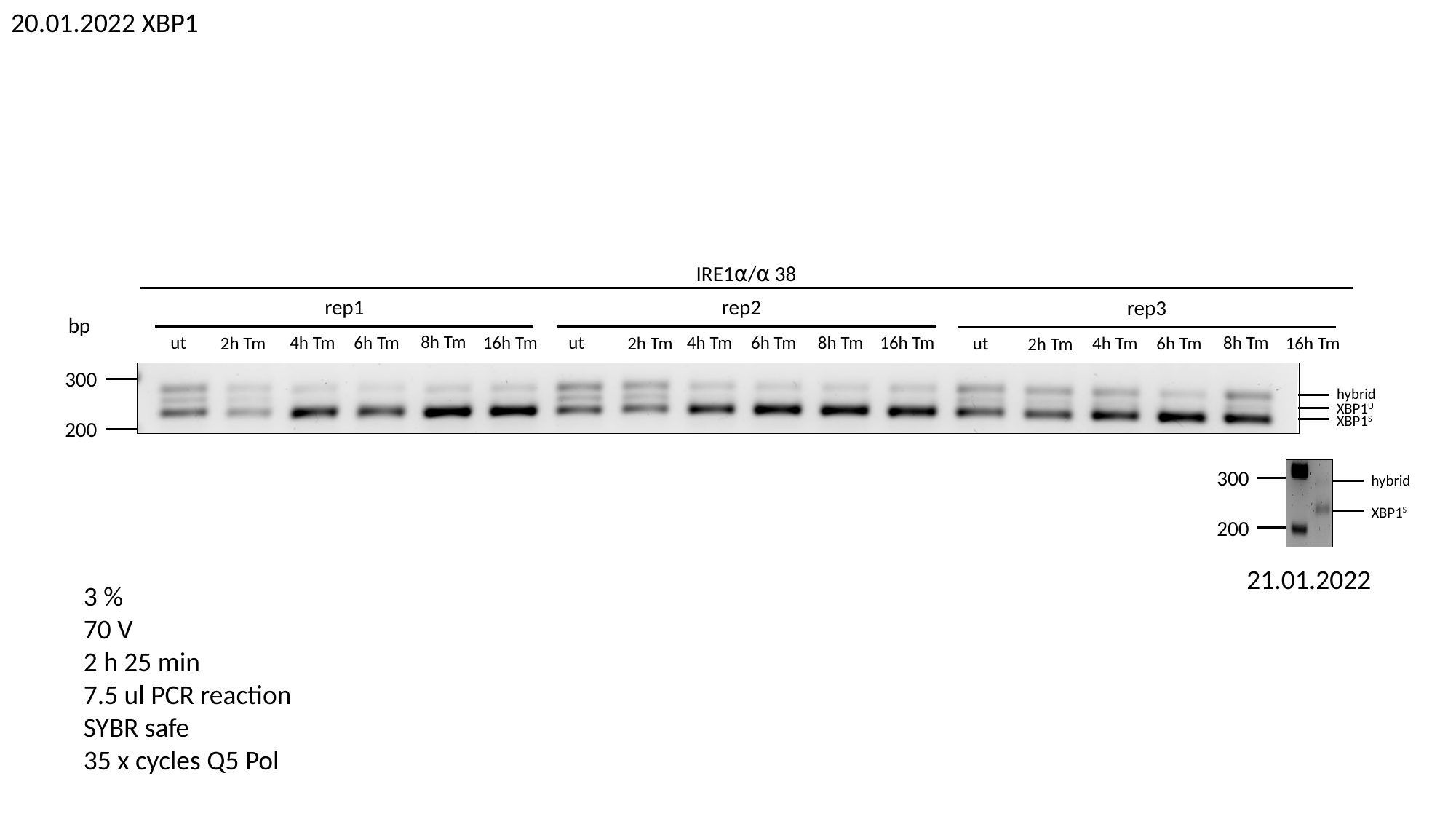

20.01.2022 XBP1
IRE1⍺/⍺ 38
rep1
rep2
rep3
8h Tm
16h Tm
8h Tm
ut
6h Tm
16h Tm
ut
6h Tm
4h Tm
4h Tm
8h Tm
16h Tm
ut
6h Tm
2h Tm
2h Tm
4h Tm
2h Tm
bp
300
200
hybrid
XBP1U
XBP1S
300
hybrid
XBP1S
200
21.01.2022
3 %
70 V
2 h 25 min
7.5 ul PCR reaction
SYBR safe
35 x cycles Q5 Pol

## Slide 3
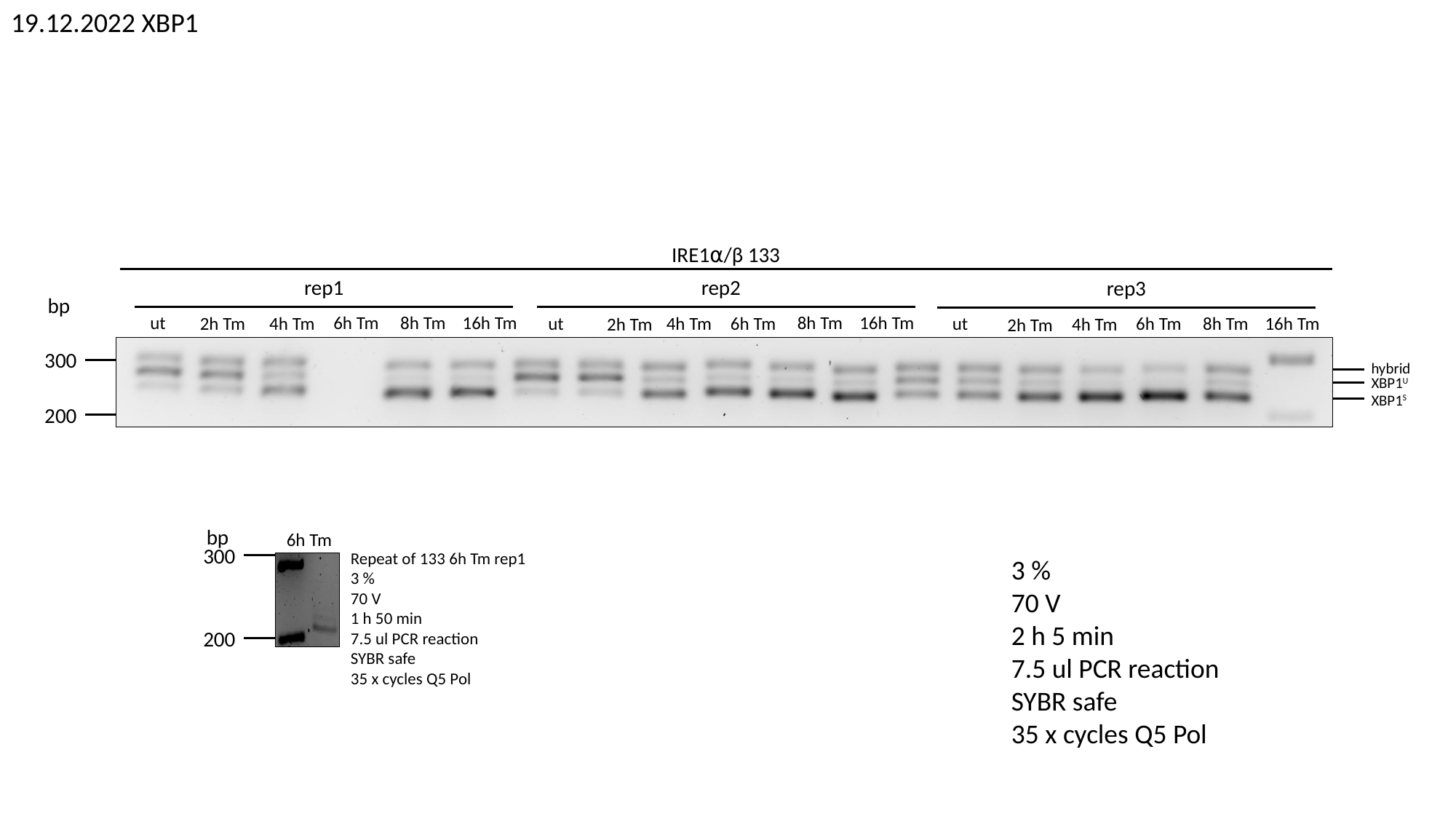

19.12.2022 XBP1
IRE1⍺/β 133
rep1
rep2
rep3
8h Tm
16h Tm
8h Tm
ut
6h Tm
16h Tm
ut
6h Tm
4h Tm
4h Tm
8h Tm
16h Tm
ut
6h Tm
2h Tm
2h Tm
4h Tm
2h Tm
bp
300
200
hybrid
XBP1U
XBP1S
bp
6h Tm
300
Repeat of 133 6h Tm rep1
3 %
70 V
1 h 50 min
7.5 ul PCR reaction
SYBR safe
35 x cycles Q5 Pol
3 %
70 V
2 h 5 min
7.5 ul PCR reaction
SYBR safe
35 x cycles Q5 Pol
200

## Slide 4
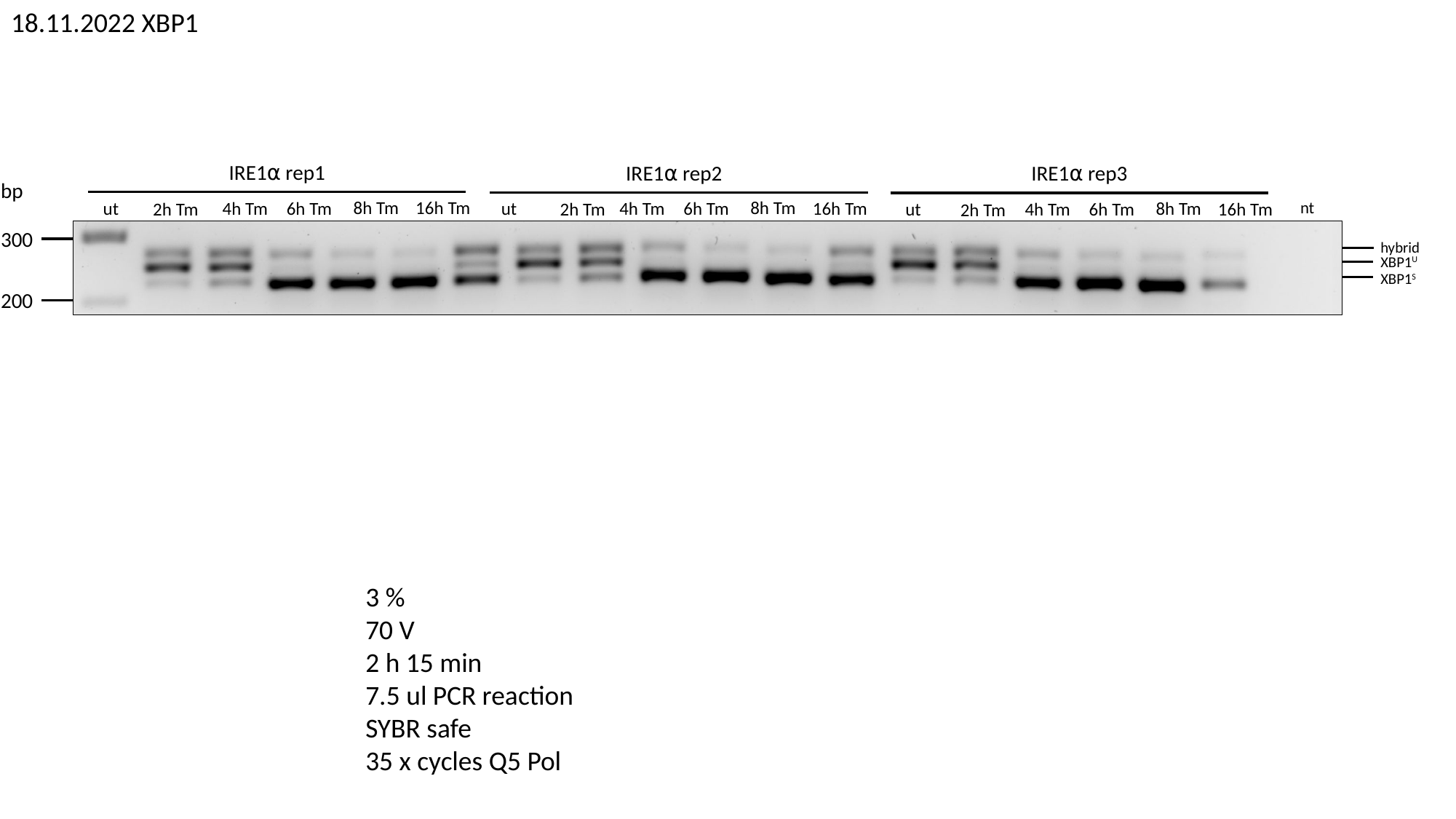

18.11.2022 XBP1
IRE1⍺ rep1
IRE1⍺ rep2
IRE1⍺ rep3
8h Tm
16h Tm
8h Tm
ut
6h Tm
16h Tm
ut
6h Tm
4h Tm
4h Tm
8h Tm
16h Tm
ut
6h Tm
2h Tm
2h Tm
4h Tm
2h Tm
bp
300
200
nt
hybrid
XBP1U
XBP1S
3 %
70 V
2 h 15 min
7.5 ul PCR reaction
SYBR safe
35 x cycles Q5 Pol
