# Supplementary material for: The IRE1β-mediated unfolded protein response is repressed by the chaperone AGR2 in mucin producing cells
Source: EMBO J. 2023 Dec 18;43(5):719–53. doi: 10.1038/s44318-023-00014-z (PMC10907699; doi:10.1038/s44318-023-00014-z)
Supplement: Supplementary file 11 — Expanded View Figures [file 44318_2023_14_MOESM11_ESM.pdf]

Expanded View Figures

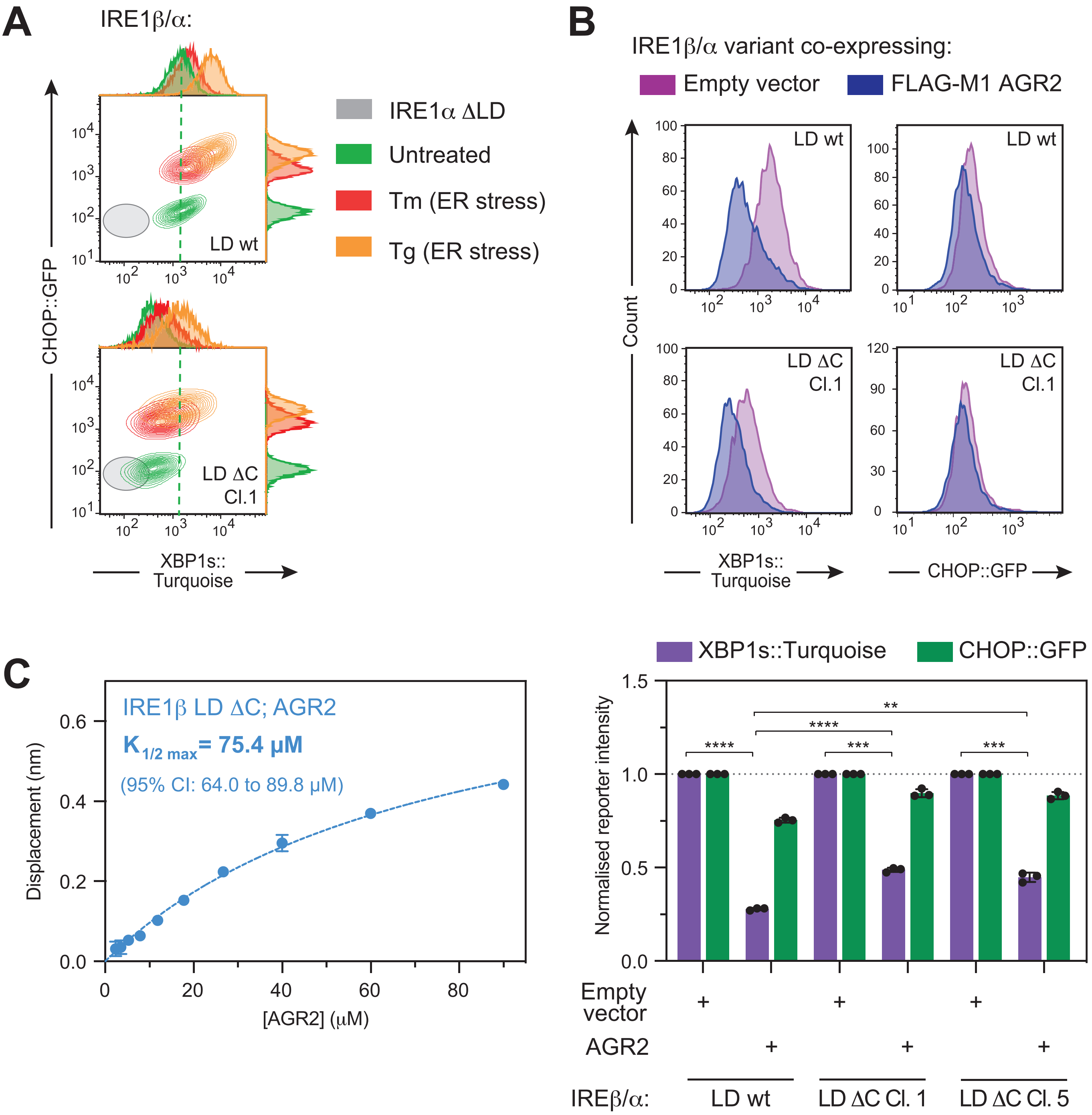

Figure EV1. IRE1β luminal domain (LD) cysteines are dispensable to its interaction with AGR2.

(**A**) Two-dimensional contour plot of CHOP::GFP and XBP1s::Turquoise signals from dual UPR reporter CHO cells expressing an IRE1β/α chimera wild-type (wt) or mutant version lacking the two cysteines of the IRE1β LD [LD ΔC Clone (Cl.) 1] from the endogenous *ERN1* locus. Where indicated cells were treated with the ER stressors tunicamycin (Tm) or thapsigargin (Tg). For reference, the signal of the IRE1α ΔLD parental cells is indicated in grey. Representative plot of three independent experiments is shown. (**B**) Upper panel: Histograms of CHOP::GFP and XBP1s::Turquoise signal from cells described in (**A**) transiently transfected with FLAG-M1-AGR2. Lower panel: Bar diagram of the mean ± SD of the indicated signal from three independent repetitions of the experiment shown in the upper panel. Statistical analysis was performed by two-sided unpaired Welch's t test and significance is indicated by asterisks (**$P < 0.01$, ***$P < 0.001$, ****<0.0001). (**C**) Bio-Layer Interferometry (BLI)-derived signal from an IRE1β LD ΔC probe as a function of concentration of interacting AGR2 fitted to a one-site specific binding function with the indicated confidence interval (CI). Shown are the mean ± SD of three independent repetitions.

© The Author(s)

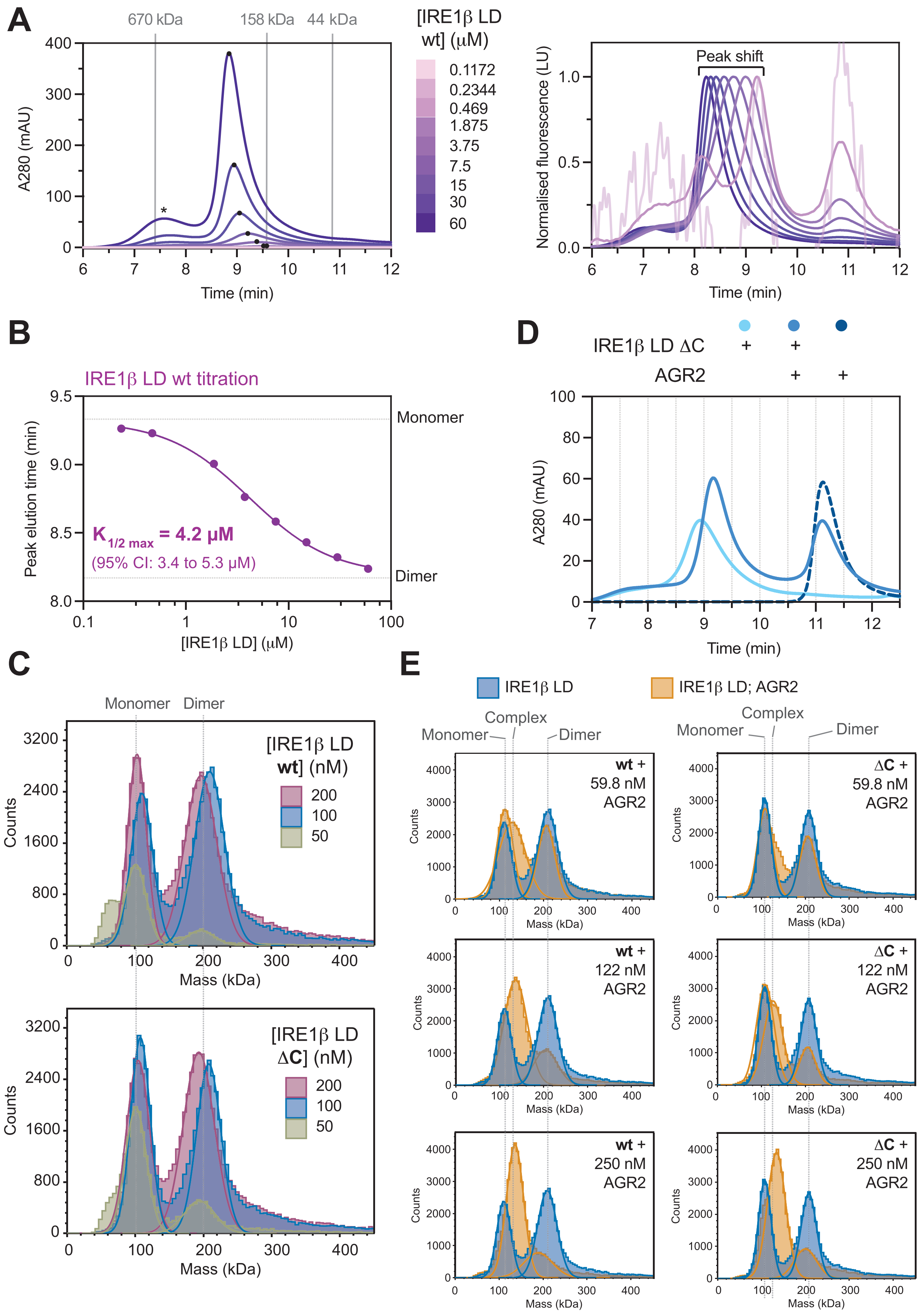
A
670 kDa
158 kDa
44 kDa
[IRE1β LD wt] (μM)
0.1172
0.2344
0.469
1.875
3.75
7.5
15
30
60
A280 (mAU)
Time (min)
Peak shift
Normalised fluorescence (LU)
B
IRE1β LD wt titration
Monomer
Dimer
Peak elution time (min)
K1/2 max = 4.2 μM
(95% CI: 3.4 to 5.3 μM)
[IRE1β LD] (μM)
D
IRE1β LD ΔC
AGR2
C
Monomer
Dimer
[IRE1β LD wt] (nM)
200
100
50
Counts
Mass (kDa)
[IRE1β LD ΔC] (nM)
E
IRE1β LD
IRE1β LD; AGR2
Complex
wt + 59.8 nM AGR2
ΔC + 59.8 nM AGR2
wt + 122 nM AGR2
ΔC + 122 nM AGR2
wt + 250 nM AGR2
ΔC + 250 nM AGR2

◀ **Figure EV2. Both, wild-type (wt) IRE1β luminal domain (LD) and a cysteine-lacking mutant (ΔC), exist in a monomer–dimer equilibrium which is shifted towards the monomeric species in presence of AGR2.**

(**A**) Left panel: Size-exclusion chromatography (SEC) elution profiles of IRE1β LD samples at the indicated concentrations. The asterisk (*) marks a high molecular weight species that is a purification artefact as it was not in equilibrium with the main peak. Right panel: Normalised fluorescence trace of the same samples, plotted against peak elution signal. (**B**) Plot of the relation of peak elution time and protein concentration of IRE1β LD from (**A**) fitted to a sigmoidal function with the indicated confidence interval (CI). (**C**) Histograms of mass photometric analysis of IRE1β LD wt or LD ΔC at the indicated concentrations. Note that the molecular mass of the monomer is 116 kDa, since IRE1β LD variants were expressed as fusion proteins with maltose binding protein and GFP. (**D**) SEC protein absorbance as in (**A**) of GFP-tagged IRE1β LD lacking its cysteines (ΔC) with and without AGR2. Representative plot of three independent experiments is shown. (**E**) Histograms as in (**C**) of 100 nM IRE1β LD wt or ΔC in presence and absence of the indicated concentrations of AGR2.

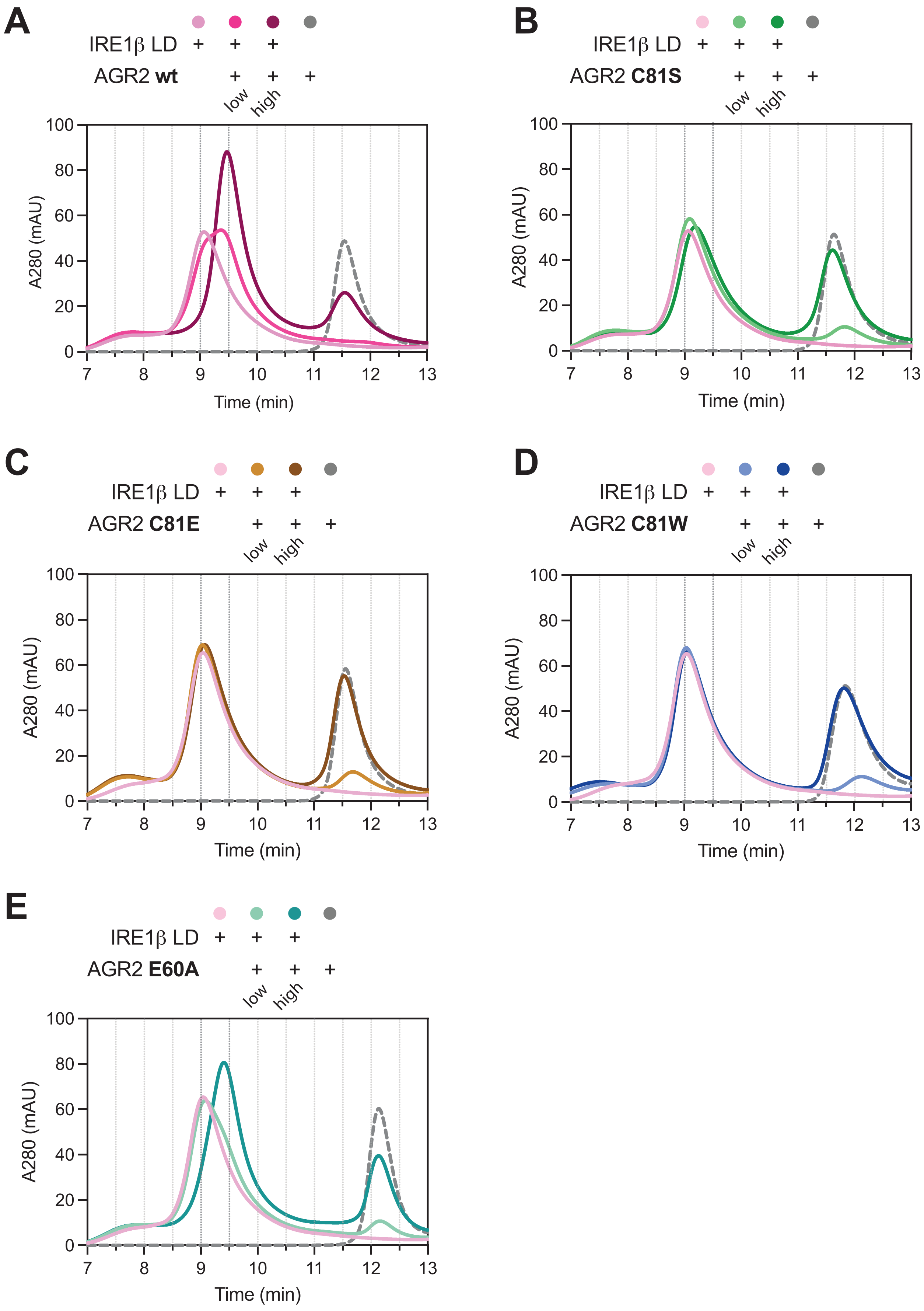
A
IRE1β LD + + +
AGR2 wt + + +
low
high
100
80
60
40
20
0
A280 (mAU)
7 8 9 10 11 12 13
Time (min)
B
IRE1β LD + + +
AGR2 C81S + + +
low
high
A280 (mAU)
Time (min)
C
IRE1β LD + + +
AGR2 C81E + + +
low
high
A280 (mAU)
Time (min)
D
IRE1β LD + + +
AGR2 C81W + + +
low
high
A280 (mAU)
Time (min)
E
IRE1β LD + + +
AGR2 E60A + + +
low
high
A280 (mAU)
Time (min)

 © The Author(s)

Figure EV3. Active-site mutations impair AGR2′s effect on the oligomeric state of IRE1β′s luminal domain (LD) in vitro.

(**A–E**) Size-exclusion chromatography (SEC) elution profiles of IRE1β LD (15 µM) incubated with 10 µM (low) or 50 µM (high) of wild-type (wt) or mutant AGR2. AGR2 E60A is a monomeric mutant previously described in (Patel et al, 2013).

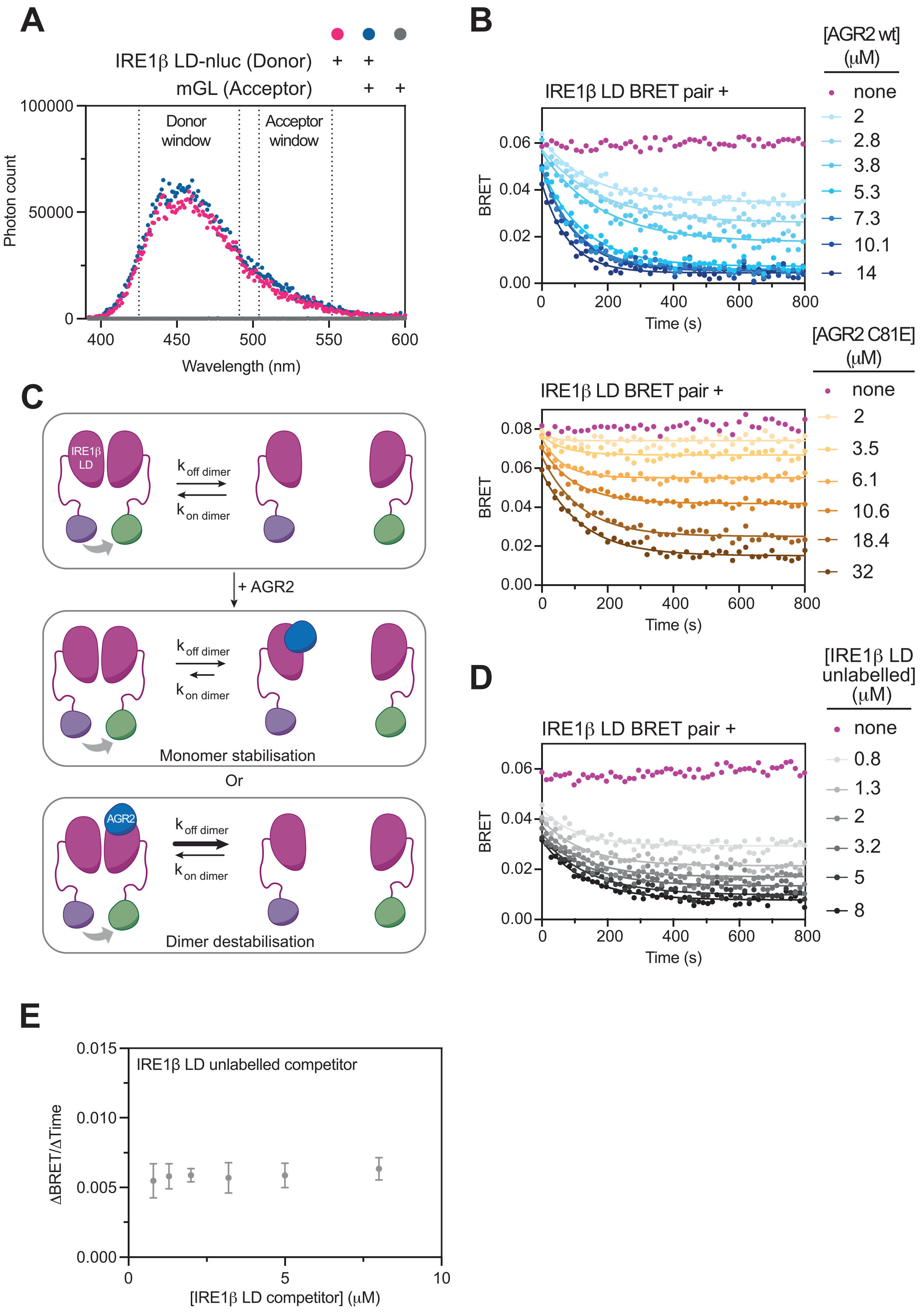
A
IRE1β LD-nluc (Donor) + +
mGL (Acceptor) + +
Photon count
Donor window
Acceptor window
Wavelength (nm)
B
[AGR2 wt] (μM)
IRE1β LD BRET pair +
none
2
2.8
3.8
5.3
7.3
10.1
14
BRET
Time (s)
[AGR2 C81E] (μM)
none
2
3.5
6.1
10.6
18.4
32
C
IRE1β LD
k off dimer
k on dimer
+ AGR2
AGR2
Monomer stabilisation
Or
Dimer destabilisation
D
[IRE1β LD unlabelled] (μM)
none
0.8
1.3
2
3.2
5
8
E
IRE1β LD unlabelled competitor
ΔBRET/ΔTime
[IRE1β LD competitor] (μM)

 © The Author(s)

Figure EV4. Kinetics of IRE1β luminal domain (LD) dimerisation and AGR2's effect on it probed by bioluminescence resonance energy transfer (BRET).

(**A**) Spectral scan as in Fig. 6B, with free monomeric Green Lantern (mGL) as an acceptor. (**B**) Plot of time-dependent change in IRE1β LD dimerisation-induced BRET following introduction of wild-type (wt) AGR2 and AGR2 C81E mutant, respectively. Traces were fitted to a one-phase exponential decay and parameters used to obtain reaction variables plotted in Fig. 6C, E. (**C**) Schematic depiction of IRE1β LD's monomer–dimer equilibrium. Two hypothetical components to AGR2 action are presented: AGR2 can bind to monomeric IRE1β LD and prevent re-dimerisation, resulting in decreased dimerisation on-rates. Whereas active destabilisation of dimeric IRE1β LD upon AGR2 binding would increase the 'off-rate' of the dimer. (**D**) As in (**B**) but following introduction of an unlabelled IRE1β LD competitor. (**E**) Plot of the rate of BRET decrease as a function of concentration of unlabelled IRE1β LD competitor. Shown are the mean ± SD of three independent repetitions.

© The Author(s)

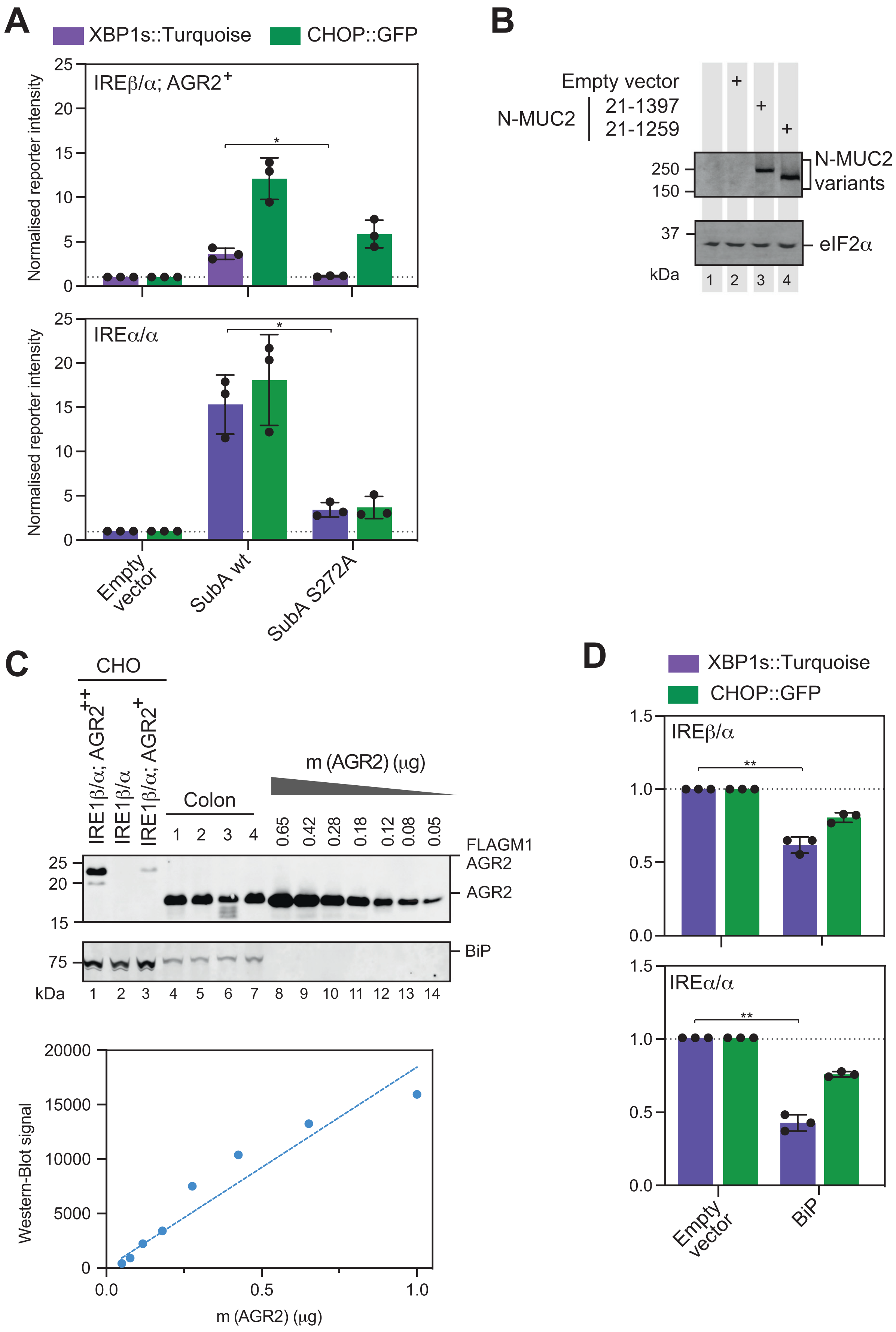
A
XBP1s::Turquoise
CHOP::GFP
IREβ/α; AGR2+
IREα/α
Normalised reporter intensity
Empty vector
SubA wt
SubA S272A
B
Empty vector
N-MUC2
21-1397
21-1259
N-MUC2 variants
eIF2α
kDa
C
CHO
IRE1β/α; AGR2++
IRE1β/α
IRE1β/α; AGR2+
Colon
m (AGR2) (μg)
FLAGM1 AGR2
AGR2
BiP
kDa
Western-Blot signal
m (AGR2) (μg)
D
XBP1s::Turquoise
CHOP::GFP
IREβ/α
IREα/α
Empty vector
BiP

 © The Author(s)

Figure EV5. Expression of MUC2 fragments and AGR2.

(**A**) Quantification of reporter signals from IRE1β/α; AGR2^{+} and IRE1α/α expressing dual UPR reporter cells transiently transfected with SubA variants (mCherry as expression marker). The inactive SubA S272A mutant was used as control. Shown are the mean ± SD of three independent repetitions. Statistical analysis was performed by two-sided unpaired Welch's *t* test and significance is indicated by asterisks (*$P > 0.1$). (**B**) Representative immunoblot of IRE1β/α; AGR2 cells transiently transfected with the indicated MUC2 variants (from Fig. 7C, D). (**C**) Upper panel shows quantitative immunoblotting (using known quantities of purified bacterially expressed AGR2 to calibrate the assay) of AGR2 the indicated CHO cell lines and mouse colonic lysates. Lower panel shows calibration curve derived from known quantities of purified AGR2. Data points were fitted to a linear function. Assuming that 50% of the mass recovered from the colonic mucosa were cells, from which 10% are goblet cells (Kim and Ho, 2010), and that the ER constitutes 10% of cell volume total volume (Alberts, 2002) the concentration of AGR2 in the goblet cell ER is about 460 μM. IRE1β/α; AGR2^{+} and AGR2 high expressing IRE1β/α; AGR2^{++} cells had a concentration of 12 μM and 140 μM of AGR2 in their ER, respectively. (**D**) Quantification of reporter signals from IRE1β/α and IRE1α/α expressing dual UPR reporter CHO cells transiently transfected with BiP (mCherry as expression marker). Shown are the mean ± SD of three independent repetitions. Statistical analysis was performed by two-sided unpaired Welch's *t* test and significance is indicated by asterisks (**$P < 0.01$).
